# A Scoping Review of the Factors That Influence Families’ Ability or Capacity to Provide Young People With Emotional Support Over the Transition to Adulthood

**DOI:** 10.3389/fpsyg.2021.732899

**Published:** 2021-10-14

**Authors:** Emily Stapley, Isabella Vainieri, Elizabeth Li, Hannah Merrick, Mairi Jeffery, Sally Foreman, Polly Casey, Roz Ullman, Melissa Cortina

**Affiliations:** ^1^Evidence Based Practice Unit, Anna Freud National Centre for Children and Families, University College London (UCL), London, United Kingdom; ^2^Riches & Ullman LLP, Carshalton, United Kingdom

**Keywords:** scoping review, transition to adulthood, young adult, social support, family, emotional support, emerging adult

## Abstract

The transition to adulthood is typically marked by changes in relationships with family members, peers, and romantic partners. Despite this, the family often maintains a prominent role in young adults’ lives. A scoping review was conducted to identify the factors that influence families’ ability or capacity to provide young people with emotional support during the transition to adulthood, and to understand the gaps in this research area. Title and abstract searches were conducted from January 2007 to February 2021 in multiple databases, including PsycINFO, MEDLINE, and Sociological Abstracts. Fifteen semi-structured interviews were also conducted with stakeholders (professionals from relevant sectors/working within this field). In total, 277 articles were eligible for inclusion in the review. Following data extraction, 19 factors were identified. Factors with the most research (more than 20 articles) included: family proximity or co-residence; mental health; sex or gender differences; and family communication. Factors with less research included: societal context; young person’s sexual orientation or gender identity; social networks; and adverse life events. Gaps in the research area were also identified, including methodological issues (e.g., lack of mixed methods and longitudinal study designs), a disproportionate focus on the parent–child relationship, and a lack of contextually situated research. Our findings indicate that future research in this area could benefit from taking an intersectional, multi-method approach, with a focus on the whole family and diverse samples.

## Introduction

The traditional markers of adulthood in Western societies, such as the completion of education, establishing a career, moving out of the family home, marriage, and becoming a parent, tend to take place at a later age now, compared to in the mid to late twentieth century (Furstenberg, 2010). In a survey of conceptions of the transition to adulthood among different age groups in the United States, Arnett (2001) found that the following characteristics were most likely to be considered important markers of the transition to adulthood: *“accepting responsibility for one’s actions, deciding on one’s beliefs and values, establishing an equal relationship with parents, and becoming financially independent”* (p. 133). In recognition of the now lengthier nature of the transition to adulthood, Arnett’s (2000, 2004, 2007) theory of ‘emerging adulthood’ captures the ‘not a teenager, but not yet an adult’ status of individuals in their late teens and twenties, defining this period as one characterized by identity exploration, possibilities, instability, self-focus, and feeling ‘in-between.’ However, critics of Arnett’s theory have commented on limitations in its cross-cultural relevance (Hendry and Kloep, 2010; Moreno, 2012), and its lack of recognition of the influence of social structures, including economic, social, and demographic factors, on the nature, timing, and ease of the individual’s transition to adulthood (Bynner, 2005; Côté and Bynner, 2008).

Indeed, Berzin (2010) found that within a sample of 8,984 youth in the United States, 25% struggled with the transition to adulthood. Demographic factors, access to resources, availability of support (such as from family or social networks), and internal characteristics (such as autonomy, motivation, and coping skills) can all influence how young people manage the transition to adulthood (Masten et al., 2004; Berzin, 2010). In the United Kingdom, the Health Foundation’s Young People’s Future Health Inquiry found that young adults (aged 22–26) identified four assets that help with the transition to adulthood: the right skills and qualifications; personal connections; financial and practical support; and emotional support (Kane and Bibby, 2018). The present study is situated within the context of the 2020/2021 phase of the Health Foundation’s Young People’s Future Health Inquiry, which seeks to further explore the fourth asset identified by young adults: emotional support (Health Foundation, 2021). Emotional support can be defined in terms of the expression of empathy, care, reassurance, and trust, in the context of having someone to talk to and a space for expressing emotions (Cohen, 2004; Kane and Bibby, 2018). Specifically, the present study examines emotional support as provided by young people’s families over the transition to adulthood, and the factors that can influence this. This topic was selected by the Health Foundation as an area in their inquiry where the scale and extent of the academic literature on this topic was currently unclear.

The transition to adulthood involves a reshuffling of one’s social relationships, as relationships with peers and romantic partners become more prominent (Lindell and Campione-Barr, 2017), and relationships with family members become voluntary to maintain (Aquilino, 2006). Peers and romantic partners can represent important sources of support for young people over the transition to adulthood (Guan and Fuligni, 2015). However, in many ways it seems that the family still maintains a significant supportive presence in young people’s lives over the transition to adulthood, with young adults often remaining dependent on their parents, at least financially and residentially, throughout their twenties (Lindell and Campione-Barr, 2017). Indeed, existing research has highlighted the role of the family as a crucial source of support for young people over the transition to adulthood, with young people appearing to draw on their families for emotional and material support over a longer period of time now, as compared to earlier generations (Oliveira et al., 2020).

However, cultural differences have also been observed in terms of the extent and prominence of the family’s role as a source of support for young people over the transition to adulthood. For example, young people in southern Europe, as compared to Northern Europe, are more likely to remain in the family home for an extended period of time and then directly transition to marriage and parenthood (Iacovou, 2002). Moreover, a study in the United States found that whereas young people from European American backgrounds reported receiving increasing parental emotional support (including seeking help, advice, and sympathy from parents) over the transition to adulthood, young people from Asian and Latin American backgrounds reported stability over time in parental emotional support (Guan and Fuligni, 2015). The value placed on family ties can also vary between cultures, for instance ‘familism,’ which emphasizes the importance and centrality of the family unit, is a value particularly endorsed within Latino, Spanish, or Hispanic cultures (Updegraff et al., 2005). Yet, across cultures, studies have also identified associations between emotional support from parents or siblings and a range of positive outcomes for young adults, including the use of more constructive coping and emotion regulation strategies (Cabral et al., 2012), greater life satisfaction (Hollifield and Conger, 2015), emotional wellbeing (Peyper et al., 2015), and higher levels of self-esteem and lower levels of depressive symptoms (Guan and Fuligni, 2015).

Despite the utility of familial emotional support over the transition to adulthood, the ability or capacity of the family to provide support for young people can be affected by multiple factors (Fingerman, 2017). Some factors may be internal to the family, for example, parental mental health or family socioeconomic status (e.g., Reising et al., 2013; Vreeland et al., 2019), and others may be external, for example, changes in economic climate or the influence of government policies (e.g., Elder, 2001; Fingerman et al., 2020). To account for this, Fingerman et al. (2020) have proposed the intergenerational systems in context (ISC) model, which considers the associations between microlevel familial processes and macrolevel societal factors and their implications for parent–child ties. Understanding the factors that can affect the ability or capacity of the family to provide emotional support for young people over the transition to adulthood has implications for policymakers and services seeking to provide support for young people and families.

To date, however, no review of the literature has been conducted specifically to map both the internal and external factors that existing research has shown can influence emotional support provision by young people’s families (broadly defined to include parents or carers, siblings, and extended family members, and families of varying structure) over the transition to adulthood. Consequently, the aim of the present study was to conduct a scoping review of the literature, alongside interviews with stakeholders working within this field, to understand what we already know about the factors influencing the family’s ability or capacity to provide emotional support for young people over the transition to adulthood, and where there are gaps in this research area.

## Methods

### Scoping Review

The Joanna Briggs Institute (JBI) methodology for scoping reviews (Peters et al., 2020) and the Preferred Reporting Items for Systematic Reviews Extension for Scoping Reviews (PRISMA-ScR; Tricco et al., 2018) guided our scoping review methodology. The primary review question was: what factors influence families’ ability or capacity to provide young people with emotional support to equip them to manage the transition to adulthood?

#### Participants

Participants included adolescents and young adults aged 12–24. Participants also included the family unit (of any structure, e.g., biological and step) and family members of any age (including parents or carers, siblings, grandparents, other extended family members, stepparents, and stepsiblings) identified by studies as contributing to, or providing, emotional support for young people.

#### Concepts

We defined the transition to adulthood (Concept 1) as a gradual process that enacts across adolescence and the twenties as young people develop cognitively, emotionally, and socially over time. Our definition of the family unit and family members (Concept 2) was not limited to any specific family structure or family member. Cohen (2004) has defined emotional support as *“the expression of empathy, caring, reassurance, and trust and provides opportunities for emotional expression and venting”* (pp. 676–677). The young people who took part in the Health Foundation’s Young People’s Future Health Inquiry described emotional support as *“having someone to talk to, be open and honest with and who supports their goals in life”* (Kane and Bibby, 2018, p. 8). It also became clear through our initial literature searches to inform our search terms that emotional support was a relatively broad, ill-defined construct in existing research, which could be construed as synonymous with such terms as closeness, intimacy, warmth, nurturance, connectedness, bonding, affection, empathy, and cohesion in family relationships. The latter are often referred to in the literature within the context of the quality of family relationships, interactions, and communication, and family member involvement, acceptance, responsiveness, and attentiveness to young people’s needs. Thus, given the exploratory nature of our review, emotional support (Concept 3) was defined very broadly as a concept that could reflect all of the above.

#### Context

As the focus of our review was on emotional support provided by the family for young people over the transition to adulthood, our search terms specified late adolescence and young adulthood as our developmental periods of interest. However, our age range included young people aged 12–24 to reflect our definition of the transition to adulthood as a gradual process that enacts across adolescence and the twenties. Studies examining the factors and processes that might enact longitudinally across adolescence and into emerging, early, or young adulthood were therefore relevant to our review. Studies were not limited to any geographical location.

#### Types of Evidence Sources

Quantitative, qualitative, and mixed methods studies and literature reviews (including peer-reviewed articles, and Masters and doctoral dissertations) available and accessible online were all eligible for inclusion.

#### Inclusion/Exclusion Criteria

Studies were included if they fulfilled all of the following criteria:

(a) The study explored the role of the family or specific family members as a source of emotional support for young people.
(b) The study explored the impact or influence of factors internal and/or external to the family on the ability or capacity of the family or specific family members to provide emotional support for young people.
(c) The young people in the study were aged 12–24. Studies that also included younger or older participants were still eligible if the age range overlapped with 12–24 years of age for the majority of the sample (within a range of 5 years for the upper age limit).
(d) The study was published in English or with an accessible English translation.
(e) The study was published from the 1st January 2007 onward (the year that the oldest participants in the review age group turned 12).

We excluded studies from our review if they focused on the experiences of young people and/or families with a physical health disorder, illness or disability, learning difficulty or disability, or neurodevelopmental disorder. Similarly, studies were also excluded from our review if they focused on evaluating the influence of a specific family support intervention. This was because our search terms did not specifically seek to identify such studies and so any identified studies were incidental rather than systematic. Given the large volume of research in these areas, such populations should constitute the focus of additional reviews.

#### Search Strategy

See Supplementary Appendix A for the final list of search terms. Title and abstract searches were conducted in the following databases for literature published between January 2007 and February 2021: PsycINFO, MEDLINE, Web of Science Core Collection, Sociological Abstracts, Sociology Database, Social Science Database, Applied Social Sciences Index and Abstracts (ASSIA), and International Bibliography of the Social Sciences (IBSS).

#### Review Process

**FIGURE 1:**
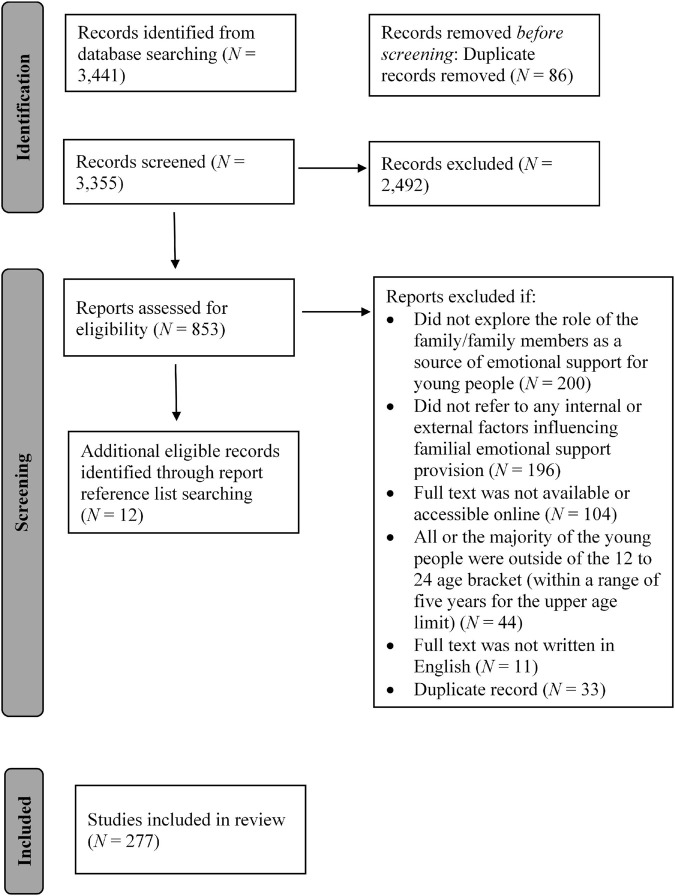
PRISMA flow diagram illustrating the flow of information and identified records at each phase of the scoping review (Tricco et al., 2016, 2018).

The Covidence software package was used to facilitate our review process, including screening and data extraction. Figure 1 presents a PRISMA flow diagram to illustrate the flow of information and identified records at each phase of the scoping review (Tricco et al., 2016, 2018). All titles and abstracts were double screened by two reviewers and those which met our inclusion criteria were taken forward for full text screening. Any discrepancies between reviewers were resolved through discussion with and review by the first author. All full texts were single screened. Relevant data were then extracted from all full text records that met the review inclusion criteria. Each article was represented by a separate data extraction form in Covidence, with the following forms of data extracted: aims or research questions; models or theories of family relations or support referenced; study design and methodology; geographical locations; sample and participants; description of emotional support provided by the family; internal or external factors that influence the ability or capacity of the family to provide emotional support for young people; study limitations; suggestions for future research.

Following data extraction, all data extraction forms were exported from Covidence into a Microsoft Excel spreadsheet. The review team then met to review the content of the spreadsheet and identify the internal and external factors influencing the ability or capacity of the family to provide emotional support for young people, which had been studied in each article. A consensus building and discussion approach was taken by the review team to group the articles under broad factor headings or themes, as identified ‘bottom-up’ by the review team through reviewing the data extraction forms together. All articles were assigned to at least one factor by the review team. Articles were grouped under more than one factor heading if the article studied more than one factor.

### Stakeholder Consultation

Professionals from relevant sectors were invited to take part in the stakeholder consultation about their perception of the factors influencing familial emotional support and gaps in the evidence base. See Supplementary Appendix B for a copy of the interview schedule. The stakeholder interviews were conducted in parallel with the scoping review. Thus, stakeholders were asked to provide their own perspectives and interpretations, without knowledge and independently of the scoping review findings. Stakeholders were identified by the Health Foundation, the Anna Freud National Centre for Children and Families, and through recommendations made by invited stakeholders. An invitation to take part in the stakeholder consultation was also put in the Centre’s newsletters.

Fifteen semi-structured interviews were conducted with 17 stakeholders (two interviews were attended by two stakeholders). All stakeholders were based in the United Kingdom. Three stakeholders were academics conducting research in the field of family relationships and/or young people’s health and wellbeing, four were involved in policymaking, four were employed by evidence-based organizations supporting young people and families, two were employed by organizations working with different types of family structures, and two provided a schools perspective.

All stakeholders gave their written informed consent to take part, including understanding that their participation was voluntary, the content of their interviews would be kept confidential, they could withdraw at any time, and anonymised findings would be published in reports or presentations. The interviews were conducted using Microsoft Teams or over the telephone and audio-recorded using an encrypted dictaphone. Interview lengths ranged from 20 to 70 min (*M* = 40.80, *SD* = 11.74). All interviews were transcribed verbatim and analyzed thematically using the NVivo 12 qualitative data analysis software package. The identities of all stakeholders have been anonymised in this report and any identifying details were also anonymised in interview transcripts.

### Public Involvement

A young adult advisor and a parent advisor at the Anna Freud National Centre for Children and Families helped us to develop our scoping review search terms and stakeholder interview schedule, and assisted with conducting the stakeholder interviews.

## Results

A total of 277 studies were eligible for inclusion in the scoping review. Summary information about these studies is reported in Table 1. Thirty-six articles (13.0%) were Masters or doctoral dissertations. As Table 1 shows, the majority of the studies were conducted in the United States (66.8%). The majority were peer-reviewed quantitative studies (81.2%), with smaller quantities of qualitative studies (11.6%), mixed methods studies (4.3%), and literature reviews (2.5%) identified. The majority of the empirical research employed a cross-sectional design (65.7%). Young people’s parents were the most researched family members (60.0%). We identified 19 factors across the 277 studies that can influence the family’s ability or capacity to provide emotional support for young people over the transition to adulthood. Supplementary Table 2 presents the full list of articles organized by factor. Definitions of the factors and examples of study findings for each factor are reported in the following subsections.

**TABLE 1:** Summary information about the 277 identified studies.

		Total number	Percentage (of total papers, *N* = 277)*
**Geographical location**			

	United States	185	66.8%
	Not specified	13	4.7%
	Canada	9	3.2%
	Israel	7	2.5%
	Germany	6	2.2%
	International/worldwide	6	2.2%
	United Kingdom	6	2.2%
	Italy	5	1.8%
	Spain	4	1.4%
	Slovenia	4	1.4%
	Portugal	3	1.1%
	Sweden	3	1.1%
	Australia	3	1.1%
	Netherlands	3	1.1%
	South Korea	4	1.4%
	Switzerland	2	0.7%
	Philippines	2	0.7%
	New Zealand	2	0.7%
	Hong Kong, SAR China	2	0.7%
	Ghana	2	0.7%
	Denmark	2	0.7%
	Iceland	2	0.7%
	Poland	2	0.7%
	China	2	0.7%
	Belgium	1	0.4%
	Kenya	1	0.4%
	Columbia	1	0.4%
	India	1	0.4%
	Taiwan	1	0.4%
	Ireland	1	0.4%
	Greece	1	0.4%
	Singapore	1	0.4%
	Turkey	1	0.4%
	Pakistan	1	0.4%
	South Africa	1	0.4%
**Study methodology**			
	Quantitative	225	81.2%
	Qualitative	32	11.6%
	Mixed methods	12	4.3%
	Cross-sectional	182	65.7%
	Longitudinal	87	31.4%
	Literature/systematic review	7	2.5%
	Meta-analysis	1	0.4%
**Family members identified**			
	Parents	166	60.0%
	Siblings	61	22.0%
	Family unit	34	12.3%
	Mothers	20	7.2%
	Grandparents	15	5.4%
	Extended family	10	3.6%
	Fathers	9	3.2%
**Factors**			
	Adverse life events	17	6.1%
	Biological factors	4	1.4%
	Ethnicity and culture	56	20.2%
	Family communication	47	17.0%
	Family proximity or co-residence	27	9.7%
	Family relationship quality	43	15.5%
	Family structure	56	20.2%
	Mental health	26	9.4%
	Parenting styles	28	10.1%
	Personality	6	2.2%
	Physical health	3	1.1%
	Sex or gender differences	50	18.1%
	Shared experiences, interests, and activities	14	5.1%
	Social networks	2	1.0%
	Societal context	8	2.9%
	Socioeconomic status	25	9.0%
	Stage of development or age of the young person	46	16.6%
	Young person’s romantic relationship status or transition to parenthood	9	3.2%
	Young person’s sexual orientation or gender identity	8	2.9%

*Some studies covered multiple criteria.

### External Factors

#### Societal Context

Eight studies were categorized to a factor called ‘societal context.’ These studies reported on trends and policies at the societal level that can influence the family as source of emotional support for young people over the transition to adulthood. Data comparisons from the United States show that young adults in the 2000s report more frequent contact, support from, and similarity in values with their parents than young adults in the 1980s and 1990s (Fingerman et al., 2012a). The transition to adulthood is now more prolonged, with young people reaching traditional adulthood milestones later now than they did 20 years ago, which contributes to greater interdependency between young adults and their parents (Fingerman et al., 2020). Advances in communication technology, societal shifts in terms of economic challenges, and the influence of public or government policies, such as health care, guardianship, or immigration policies, have all contributed to this (Fingerman, 2017). For example, in terms of economic challenges, the economic recession, which began in 2007, has been a key event shaping parent–child ties and contributing to increases in co-residence between young adults and their parents in such countries as the United States and the United Kingdom (Fingerman, 2017; Fingerman et al., 2020). Moreover, the Covid-19 pandemic is another major event currently contributing to economic difficulty and uncertainty for young people and their families, which will have implications for families’ experiences of the transition to adulthood (Oliveira et al., 2020).

#### Social Networks

Two studies examined how young people’s and the family’s ‘social networks’ are associated with the family’s provision of emotional support for young people over the transition to adulthood. A quantitative study suggested that if social networks (including friends or other adults, e.g., teachers) have prominence in young people’s lives then the family may be less central as a source of support, or social networks may compensate for diminished family support (Reis et al., 2009). A qualitative study found that the ability of foster carers to provide support for young people was influenced by their relationships with supportive others in their environments (e.g., social workers); foster carers were better able to support young people when they felt that they were part of a team of professionals (Munford and Sanders, 2016). Foster carers also helped young people to connect with their immediate and extended family of origin, and foster carers’ own families became part of young people’s support networks (Munford and Sanders, 2016).

### Family Process Factors

#### Parenting Styles

‘Parenting styles’ were investigated as an influencing factor in 28 studies, including authoritative parenting (versus authoritarian, permissive, and neglectful parenting), autonomy-supporting parenting (versus helicopter parenting or controlling behavior), and parental differential treatment (PDT) of siblings. Overall, studies examining this factor have found that parenting styles can affect the quality of family relationships. For instance, authoritative parenting, which is characterized by high levels of warmth, responsiveness, and support, and moderate levels of control, appears to be positively associated with the quality of the parent–child relationship and perceived family support over the transition to adulthood (Shenaar-Golan and Goldberg, 2019), as does autonomy-supporting parenting (Jung et al., 2020). High levels of parental care are associated with positive sibling relationships, whereas high levels of parental control (accompanied by low levels of care) are associated with more negative sibling relationships in young adulthood (Portner and Riggs, 2016). PDT of siblings has also been found to negatively affect sibling relationship quality. For example, perceived favoritism by parents is associated with poorer quality sibling relationships, sibling rivalry, lower levels of sibling warmth, and less sibling intimacy (e.g., Jensen, 2012; Gozu, 2017; Woo, 2020).

#### Family Relationship Quality

Forty-three studies examined associations between ‘family relationship quality’ and familial emotional support provision. Research has shown that parents support their young adult children when they have good relationships with them, when they find it rewarding to help them, and when they share values (Fingerman et al., 2012b; Fingerman, 2017). In turn, parental support is received more positively from young adults in the context of parent–child relationships characterized by warmth and high positive regard (Fingerman et al., 2013b). Studies have also looked at attachment and how this affects familial relationships (e.g., Ghali, 2010; Tibbetts and Scharfe, 2015; Alavi et al., 2020). For example, a study in Italy found that secure attachment with mothers and fathers was associated with high levels of warmth and low conflict in sibling relationships (Ponti and Martina, 2019).

Two studies have found that higher conflict between young people and their parents is associated with less perceived familial support (Bahrassa, 2013; Galambos et al., 2018). Perpetration of sibling aggression (though not sibling conflict) has been found to predict lower levels of sibling warmth, involvement, and emotional support 4 years later (Tucker et al., 2019). Several studies have also reported that interparental conflict and marital dissatisfaction have a detrimental impact on the parent–child relationship, including provision of less parental emotional support for young people, independently of parental romantic relationship status (divorced or married; e.g., Fabricius and Luecken, 2007; Frank, 2007; Riggio and Valenzuela, 2011). This association extended to sibling relationships too in one study, whereby higher levels of perceived parental marital satisfaction predicted closer and more communicative sibling relationships (Milevsky, 2019).

Studies have also examined the influence of young people’s levels of individuation and autonomy on familial emotional support provision. Over the transition to adulthood, emotional autonomy from parents appears to be linked to lower levels of warmth in the parent–child relationship, whereas other forms of autonomy, such as autonomy of ‘voice’ and in making social choices, appear to be linked to higher levels of warmth (Zimmer-Gembeck et al., 2011). It has also been found that freedom from inner conflict about individuation in emerging adulthood (e.g., guilt and anger around expressing one’s individuality with one’s parents) predicts more satisfying relationships with parents, but higher levels of functional independence are associated with less satisfying relationships with parents (Mendonca and Fontaine, 2013).

Relatively few studies have examined grandchild-grandparent relationship quality in the context of familial emotional support provision over the transition to adulthood. Studies have found that the level of closeness in grandparents’ relationships with their young adult grandchildren is associated with: the closeness of parents’ (particularly mothers) relationships with both the grandparents and the young adults (Monserud, 2008); physical proximity to grandparents (Roos et al., 2019; Wise and Onol, 2020); grandparent gender – with closer relationships typically found with grandmothers (Wise and Onol, 2020); contact frequency, medium, and quality (Wise and Onol, 2020); and the level of affectionate communication by grandparents (Mansson et al., 2017).

#### Shared Experiences, Interests, and Activities

Fourteen studies were categorized to a factor called ‘shared experiences, interests, and activities.’ In general, studies suggest that shared experiences, including shared interests (e.g., music), family activities (e.g., mealtimes, special celebrations, and quality time), life events (e.g., college attendance), and similar values, traits, and characteristics can have a positive impact on the family unit, parent–child and sibling relationships, and familial emotional support provision (e.g., Hair et al., 2008; Boer and Abubakar, 2014; Maximo and Carranza, 2016; Bayly and Bumpus, 2020; Ponti and Martina, 2020).

#### Family Communication

Forty-seven studies examined ‘family communication’ as a factor influencing familial emotional support provision over the transition to adulthood. Fingerman and colleagues concluded in their reviews of the literature that contact between young adults and their parents has increased over the past 25 years, likely facilitated by the widespread use of technology and changing economic and social conditions (e.g., Fingerman et al., 2013a, 2020; Fingerman, 2017). Synchronous communication methods, communication technologies, multiple communication channels, and the ability to hear a family member’s voice have all been found to have a positive impact on relational quality, intimacy, affection, and satisfaction (Lindell et al., 2015; Hessel and Dworkin, 2018). Phone and online contact methods enable families to maintain togetherness even when geographical distance increases, such as due to young people attending college or moving out of the family home (Marchant and O’Donohoe, 2014; Bacigalupe and Brauninger, 2017; Yu et al., 2017; Yang, 2018).

Studies have consistently suggested that a ‘conversation’ orientation within the family, whereby family members feel free to express their views, predicts perceptions of greater bonding within families (Schrodt et al., 2007; Ghali, 2010; Scruggs and Paul, 2020). Siblings who engage in ‘confirming’ (i.e., supporting each other’s endeavors, respecting viewpoints, and giving undivided attention) and ‘challenging’ (i.e., pushing each other to excel, and challenging or defending ideas) modes of communication are more likely to experience closeness and relational satisfaction in their relationships with each other (Phillips and Schrodt, 2015a). Young adults who report moderate to high ‘co-rumination’ (i.e., the interpersonal consideration of one’s problems) with parents and siblings tend to experience high levels of social support and bonding with those relational partners (Ames-Sikora et al., 2017).

Trust has been highlighted as a key aspect of young people’s relationships with their parents and siblings, influencing relationship intimacy and satisfaction, and young people’s willingness to disclose issues (Di Tunnariello, 2016; Lindell and Campione-Barr, 2017). Deterding (2010) found that young adult siblings reported disclosing private information to each other as a way of maintaining relational bonds. Adolescent disclosure is also associated with greater parental warmth and relational closeness perhaps due, for example, to the greater shared sense of connection that follows from this (Padilla-Walker and Daye, 2019). In turn, parental openness and disclosure also predict increased relational closeness with emerging adults (Donovan et al., 2017).

### Family Organizational Factors

#### Family Proximity or Co-residence

Twenty-seven studies were categorized to a factor called ‘family proximity or co-residence.’ Family proximity was defined geographically, and co-residence was defined as living in the same household. Overall, studies examining the influence of family proximity or co-residence on familial emotional support provision have yielded mixed findings. Approximately half of the studies identified co-residence or proximity as a positive factor associated with better relationship quality, increased levels of support from, or emotional closeness with family members (e.g., Fabricius and Luecken, 2007; Fingerman, 2017; Fingerman et al., 2017; Kim et al., 2020; Wise and Onol, 2020). Young people who live in close proximity to family members may have more opportunities to seek and receive support from family members (Kim et al., 2020). Yet, some studies found that moving out of the parental home led to increased closeness with, stronger bonds with, or support from family members (e.g., Brooks, 2015; Galambos et al., 2018; Hamwey et al., 2019). Increased distance can improve the quality of family relations by reducing family conflict (Whiteman et al., 2011; Milevsky and Heerwagen, 2013; Brooks, 2015). Other studies found no association between co-residence or proximity and family relationship quality (Conger and Little, 2010; Petree, 2014; Sulimani-Aidan et al., 2017). While studies have often examined this factor in the context of the young person moving out of or remaining resident in the family home, several studies also examined this factor in the context of parental divorce, in terms of one of the parents (usually the father) moving out of the family home (e.g., Fabricius and Luecken, 2007; Frank, 2007; Feistman et al., 2016).

#### Family Structure

Fifty-six studies examined whether and how ‘family structure’ can influence familial emotional support provision over the transition to adulthood. These studies focused on families with divorced or separated parents, stepfamilies, adoptive families, assisted conception families, foster care families or families with a child in care, and family size or the young person’s position in the family. Regarding family size, research has shown that parents with more children generally provide less support to each child, in comparison to parents with fewer children (Fingerman et al., 2009). Research examining sibling order has found that younger children receive more of every type of support from their parents than their older siblings (Fingerman et al., 2009), and that first-borns also find themselves providing sibling care and assuming parental roles toward their younger siblings, especially when the age gap is large (Milevsky and Heerwagen, 2013; Wu et al., 2018).

Findings have been mixed with regard to the impact of adoptive family status. One study found lower reported feelings of closeness within adoptive families than non-adoptive families (Walkner and Rueter, 2014), while another found that when adopted emerging adults feel more positively about their adoption, they report higher levels of closeness with their adoptive mothers (Walkner-Spaan, 2016). Two studies found that conception via assisted reproduction (IVF or donor insemination) had no negative impact on levels of parent–child warmth from the perspective of both parents and children (Golombok et al., 2009; Owen and Golombok, 2009). Research with young people in care has found that ties with carers (e.g., foster carers and professional figures), rather than parents, are associated with their perceptions of support (Won, 2009; Sulimani-Aidan, 2019).

There is broad agreement in the literature that exposure to parental divorce or separation is associated with less perceived parental emotional support, poorer quality parent–child relationships, and lower levels of relational closeness over the transition to adulthood (e.g., Young and Ehrenberg, 2007; Bertogg and Szydlik, 2016; O’Mara and Schrodt, 2017). Father–child relationships appear to be more vulnerable to the negative effects of divorce or separation than mother–child relationships (e.g., Nicholson, 2007; Riggio and Valenzuela, 2011; Lee, 2019). Indeed, fathers are more likely than mothers to become the non-residential parent in the event of a divorce or separation (Miller, 2010). Peters and Ehrenberg (2008) found that young adults with divorced parents perceived lower levels of father involvement and nurturance, and less contact with their fathers, than young adults with married parents.

On the other hand, Scott (2010) found that adolescent offspring were closer to their non-resident fathers following their parents’ divorce when their mothers had remarried. Scott (2010) suggested that offspring may seek support from their biological fathers when adjusting to life in a new stepfamily. On average, stepparents have been found to be less involved with and to feel less obligated to offer support to their stepchildren, as compared to biological or adoptive parents (Fingerman, 2017). Factors influencing stepparent-stepchild relationships include the quality of the relationship between one’s biological parent and stepparent (King and Lindstrom, 2016; Jensen and Lippold, 2018), the length of time that stepfamilies have been together (King and Lindstrom, 2016), and the amount and quality of time that the stepparent and stepchild spend together (Ganong et al., 2011).

Parental divorce or separation can also have a negative effect on emerging adults’ sibling relationships, with studies reporting lower levels of closeness and warmth between siblings in divorced families, compared to those in non-divorced or separated families (Conger and Little, 2010; Nitzburg, 2013; Milevsky, 2019). Yet, in a qualitative study of young adult women’s experiences of parental divorce, Reed et al. (2016) found that participants most often described turning to their siblings as a source of support during their parents’ divorce, whereas comparatively few participants felt that discussing their needs with their parents during this time was helpful.

#### Young Person’s Romantic Relationship Status or Transition to Parenthood

Nine studies explored how a ‘young person’s romantic relationship status or transition to parenthood’ can affect familial emotional support provision. Two studies found that parental support (including emotional support, listening, availability, advice, and companionship) decreases when their children are married (Fingerman et al., 2016b; Kim et al., 2020). However, another study identified no differences between non-dating and dating young adults in terms of their perceptions of emotional support from their parents (Lanz and Tagliabue, 2007). Yet, Shenhav (2018) found that young adults’ perceptions of parental approval of their romantic relationships were significantly associated with higher satisfaction with and closeness in their relationships with their parents. Only a small number of studies were identified that examined young people’s transition to parenthood as a factor influencing familial emotional support provision. For example, Updegraff et al. (2018) found that within a sample of Mexican-origin adolescent mothers in the United States, earlier timing of the transition to parenthood was associated with greater stability over time in familism, which was in turn positively associated with levels of mother–child relationship warmth and social support.

### Family Stressors

#### Mental Health

‘Mental health’ as a factor influencing familial emotional support provision was explored in 26 studies. Mental health issues examined by studies included depression, emotional distress, substance use, and schizophrenia. Overall, the majority of the studies reported that the presence of mental health issues (typically depression) in either the young person or the parent negatively influences the young person’s perception of the level and effectiveness of familial emotional support, the ability of the parent to provide effective emotional support, and the quality of the parent–child relationship (e.g., Needham, 2008; Chung et al., 2009; Moberg et al., 2011; Kim, 2012; Roche et al., 2016). Studies investigating how having a sibling with mental health issues affects sibling emotional support were comparatively scarce and showed mixed results. One study reported that poor sibling mental health negatively affects levels of perceived warmth (Watson, 2019). Yet, another study reported that relationships were strong between siblings, despite the challenges of having a sibling with a diagnosed mental health condition (Jacoby and Heatherington, 2016).

#### Physical Health

We excluded studies focusing on the experiences of young people and/or family members with a physical health disorder, illness, or disability. Therefore, the three studies captured in relation to the ‘physical health’ factor referred to non-specific physical health conditions. These studies found that poor physical health – whether of the young person or another family member – was associated with less relational closeness in family relationships (Bertogg and Szydlik, 2016; Roos et al., 2019; Kim et al., 2020).

#### Adverse Life Events

Seventeen studies examined the influence of ‘adverse life events’ on familial emotional support provision, including the impact of parental death, parental incarceration, abuse, and childhood trauma. Evidence shows that losing a parent in childhood or adolescence because of death or physical distance (e.g., due to incarceration) can result in decreased family cohesion (Birgisdottir et al., 2019), less closeness in the parent–child relationship (Scott, 2010), and less parental support (Nylander et al., 2018), but greater reliance on extended family members, such as grandparents, aunts, and uncles, for support (Benson and Bougakova, 2018; Nylander et al., 2018). Parental violence or abuse and childhood polyvictimization can also negatively affect the quality of parent–child relations later in life (e.g., Barnes et al., 2016; Allbaugh, 2018; Duval et al., 2019). Moreover, adverse life events, such as parental death or parental history of trauma, can lead to additional difficulties, such as deterioration in family members’ mental health or financial status, which can then in turn affect their ability to provide emotional support for young people (Berman et al., 2015; Gamache Martin, 2018; Murry, 2019).

### Individual Factors

#### Personality

Six studies were categorized to a factor called ‘personality.’ These studies examined the influence of personality traits on familial emotional support provision. For example, in a sample of 674 young adults, Zupancic and Kavcic (2014) found that extraversion and agreeableness predicted stronger connectedness (communication, mutual understanding, respect, and trust) and higher levels of support-seeking from parents (including approval, help, and advice), conscientiousness was negatively related to levels of perceived parental intrusiveness and fear of disappointing parents (whereas neuroticism was predictive of the oppositive pattern), and openness was associated with self-reliance. Gozu (2017) found that young adults who scored highly in terms of openness, conscientiousness and agreeableness, and low on neuroticism, were more likely to report having better relationships with their siblings. Finally, in a qualitative study, Roos et al. (2019) found that perceptions of grandparents’ personality (e.g., empathetic vs. rigid) influenced whether young adult women desired closeness with, or distance from, their grandparents.

#### Biological Factors

Four studies focused on ‘biological factors’ influencing familial emotional support provision. For example, a study in the Netherlands found that there was a specific neural activation in the medial prefrontal cortex (mPFC) in young people who positively evaluated their mother’s traits, which suggests a possible neural indicator of the quality of young people’s relationships with their mothers (Van der Cruijsen et al., 2019). A study in the United States found that higher levels of waking cortisol, a hormone associated with stress, were related to stronger parental attachment, positive parenting, and bonding in a sample of adolescents examined over 6 years (Shirtcliff et al., 2017).

#### Young Person’s Sexual Orientation or Gender Identity

Eight studies were categorized to a factor called ‘young person’s sexual orientation or gender identity.’ The majority of studies reported diminished familial emotional support in the context of a young person’s minority sexual orientation or gender identity. For example, lesbian and bisexual women report lower levels of parental support (in the form of closeness, enjoying each other’s company, and receiving love and warmth) than heterosexual women, and gay men report lower levels of parental support than bisexual and heterosexual men (Needham and Austin, 2010). Platt et al. (2020) found that cisgender college students report significantly more family support (including emotional help and support) and less family distress compared to transgender and gender-non-conforming students. In a qualitative study, gender dysphoria was found to negatively affect the parent–child relationship, as young people isolated themselves from their parents for fear of being judged or rejected (Littman, 2018).

#### Stage of Development or Age of the Young Person

Forty-six studies reported on how familial emotional support provision can be affected by the ‘stage of development or age of the young person.’ In general, findings regarding the influence of this factor have been inconsistent. Some studies have identified a decrease in parent–child relationship quality, closeness, communication quality, and support during the transition to adulthood, as compared to earlier life (e.g., Zupancic et al., 2014; Bowles and Hattie, 2015; Galambos et al., 2018), some have reported improvements (e.g., Jimenez, 2008; Morgan et al., 2010; Guan and Fuligni, 2015), and others have found no significant changes (e.g., Proulx and Helms, 2008; Moilanen and Raffaelli, 2010; Lindell, 2019).

Among studies that have investigated changes in sibling relationships across development, the majority have reported increased, improved, or stable emotional support, communication, and warmth among siblings during the transition to adulthood, as compared to earlier life (e.g., Halliwell, 2016; Killoren et al., 2016; Oliveira et al., 2020). On the other hand, research has also identified a decrease in support from grandparents over time, finding that grandparents are perceived as an important support source primarily in preadolescence (Spitz et al., 2020). However, studies have also shown that while contact between young people and their families (including grandparents) can decline over the transition to adulthood, their levels of emotional closeness remain stable (Neyer and Lehnart, 2007; Wetzel and Hank, 2020).

### Sociodemographic Factors

#### Ethnicity and Culture

Fifty-six studies were categorized to a factor called ‘ethnicity and culture,’ which examined familial emotional support provision in the context of cultural or ethnic group differences and similarities. We note that with regard to this factor, differences observed between ethnic groups do not reflect ethnicity as a causal factor behind such differences. Rather, any differences may in fact reflect the structural inequalities and discrimination that individuals from different ethnic groups face, as well as differences in cultural values, norms, and family life. Around 80% of the studies that we identified in relation to this factor were conducted in the United States.

Studies have explored ethnic and cultural differences within the same country. For instance, in a study of United States college students’ perceptions of emotional support from extended family, Budescu and Silverman (2016) found that Asian American students reported significantly lower levels of emotional support than both European American and African American students. However, Asian American students also reported that they had fewer extended family members living near them (Budescu and Silverman, 2016). In another United States-based study, Hardie and Seltzer (2016) found that first-generation Hispanic immigrant young adults and non-immigrant Black young adults were more likely to turn to their parents for relationship advice than other ethnic groups. In addition, Black and Hispanic young adults of all immigrant generations were more likely to live with their parents than White young adults (Hardie and Seltzer, 2016). Murry (2019) found that the degree of exposure to racial discrimination affected African American parents’ provision of emotional support for their adolescent children. Specifically, parents with low-level exposure to racial discrimination provided more emotional support compared to those with high-level exposure to racial discrimination (Murry, 2019).

Some studies have examined associations between immigration status and familial emotional support provision. For example, Dillon et al. (2012) found that participants with documented immigration status in the United States (many of whom were Cuban) reported higher levels of family cohesion than participants with undocumented immigration status (many of whom were Central American). The lower levels of family cohesion among undocumented immigrants may be due to such factors as the high degree of economic instability and separation that these families experience compared to documented immigrants (Dillon et al., 2012). Research has shown that young immigrant adults perceive higher levels of relationship strength and bonding when their parents or siblings share their immigration status (e.g., documented or undocumented) (Morales et al., 2020). However, higher levels of language brokering (children translating for their parents) appear to be associated with lower levels of perceived parental support, as mediated by parental praise (Guan and Shen, 2015). Being born in a country different to both immigrant parents can lead to intergenerational acculturative dissonance, which can negatively affect parent–child closeness (Maru, 2021).

Studies have also explored ethnic and cultural differences in familial emotional support provision between different countries. For instance, Mansson and Sigurdardottir (2019) found that young adult grandchildren in the United States reported more perceived love, caring, and celebratory affection from their grandparents, as compared to Danish, Icelandic, and Polish young adult grandchildren. Alavi et al. (2020) identified significant differences in associations between family adaptability or cohesion and close attachment across the Western and non-Western country groupings in their sample. They suggested that such differences may be the result of a greater emphasis on collectivistic cultural values in non-Western countries, whereby family members’ interdependency has a positive influence on family relationship closeness (Alavi et al., 2020).

The concept of familism has been consistently found to be associated with high levels of acceptance, intimacy, closeness, and warmth in young people’s relationships with their parents and siblings (e.g., Baham, 2009; Killoren et al., 2015; Streit et al., 2018; Updegraff et al., 2018; Padilla et al., 2020). Indeed, studies have found that young people who have similar cultural values and beliefs, ethnic backgrounds, and religious beliefs to their family members report higher family cohesion, support, and closeness in their relationships with their parents and siblings (e.g., Tanaka, 2016; Hwang et al., 2019; Sheu, 2019; Atkin and Jackson, 2020). In a qualitative study, Atkin and Jackson (2020) found that parents with different ethnic characteristics to their young adult children were perceived by their children as being unable to understand the discrimination that they had experienced, as they could not relate to their experiences. This negatively affected the perceived quality of the support provided by their parents, especially if it involved them giving advice about how to handle discrimination (Atkin and Jackson, 2020). However, knowing that their parent was making an effort to support them, even if their advice was not very helpful, was nonetheless perceived as important (Atkin and Jackson, 2020).

#### Sex or Gender Differences

Fifty studies explored ‘sex or gender differences’ in the context of familial emotional support provision. Across studies, there is a general trend showing that women report higher levels of familial support, relationship quality, connectedness, and warmth than men (e.g., Beyers and Goossens, 2008; Moilanen and Raffaelli, 2010; Galambos et al., 2018; McKinney et al., 2018; Kim et al., 2020). Moreover, young people report receiving higher levels of support from mothers than from fathers, and mothers tend to be perceived as having a more supportive role than fathers (e.g., Umaña-Taylor et al., 2012; Zupancic et al., 2012; Mendonca and Fontaine, 2013; Galambos et al., 2018). Young people also report experiencing greater closeness, support, and warmth in their relationships with sisters than with brothers (e.g., Finzi-Dottan and Cohen, 2011; Cassidy et al., 2014; Tibbetts and Scharfe, 2015). Finally, studies with grandparents have shown that maternal grandmothers provide the highest levels of emotional support for young people (Wise, 2008; Gray et al., 2019).

#### Socioeconomic Status

Twenty-five studies examined the role of family ‘socioeconomic status’ (SES), including income, employment status, and education level, in influencing familial emotional support provision. Research suggests that young people in lower SES families are more likely to receive less support from their parents during young adulthood, due to such factors as lack of resources and higher stress (e.g., Fingerman et al., 2015; Hartnett et al., 2018; Quan, 2020). Studies have shown that the degree of closeness, openness, and quality of young people’s relationships with their parents is associated with the amount of financial support that their parents provide for them or with their degree of financial dependence on their parents (e.g., Johnson, 2013; Lindell, 2019; Lindell et al., 2020). On the other hand, some studies have found no influence of SES on familial emotional support provision and relationship quality (Melby et al., 2008; Fingerman et al., 2009; Roksa, 2019).

Studies have shown mixed findings with regard to the influence of young people’s employment status on familial emotional support provision (e.g., Roy et al., 2010; Bertogg and Szydlik, 2016; Kim et al., 2020). In terms of parental employment, Lim (2012), for example, found that parental absence at home (due to long working hours) affected parents’ emotional closeness with their children. Regarding young people’s education level, Fingerman et al. (2012c) found that college students received more support from their parents than non-students, and Brooks (2015) found that attending college can contribute to improved closeness with parents and siblings. Higher parental education level has been found to be associated with supportive parenting and bonding in the parent–child relationship (Melby et al., 2008; Neves et al., 2019).

### Stakeholder Consultation Findings

Additional factors influencing familial emotional support provision identified by stakeholders included: the role of other significant adults in the young person’s life (e.g., teachers); families’ degree of access to resources to facilitate their provision of emotional support (e.g., charities and organizations supporting families); family members’ emotional intelligence and confidence in providing emotional support; and families’ relationships with statutory services (e.g., child and adolescent mental health services; CAMHS).

Stakeholders described a need within the research field for up to date statistics on both ‘traditional’ and ‘non-traditional’ family structures, for example kinship carers, and access to data that clearly shows the breadth and range of family structures, in order to further our understanding of the heterogeneity of families and move beyond the concept of the nuclear family. Stakeholders highlighted the importance of consideration of the impact of social change on familial provision of emotional support for young people. They recognized that life for young people is very different today compared to previous generations, with discussion of the “*digital revolution,”* for example, which has resulted in significant changes in communication between generations (e.g., use of social media).

Stakeholders identified several voices that they felt were missing from the current evidence base. This included not only the voices of siblings, grandparents, aunts and uncles as sources of emotional support for young people outside of their parents, but also the voices of parents and young people themselves on what they need or want in terms of support, and what helps. Stakeholders also felt that there was a need for research to further our understanding of familial emotional support in the context of disadvantaged or minority groups, such as young people or parents who identify as LBGQT+, or families from minority ethnic backgrounds.

Another theme across stakeholders’ suggestions for future research was the need for more of a focus on translational and implementation research. Stakeholders’ suggested next steps were to take learning from research conducted so far and put in place interventions, services, or programs stemming from this research that support the family’s ability to provide emotional support for young people, with a focus on working with families to understand what would work for them in their particular contexts.

Finally, no academic studies exploring the impact of the Covid-19 pandemic on the family’s ability or capacity to provide emotional support for young people over the transition to adulthood were identified through our scoping review. However, stakeholders suggested that there is a need for longitudinal research investigating the longer-term impacts (and the mechanisms behind this) of the Covid-19 pandemic for young people and families, and their implications for familial emotional support provision. Stakeholders commented that the pandemic’s impact on young people’s future financial independence, employment, and access to housing could affect the level of support needed from their families.

## Discussion

A scoping review of the literature yielded 19 factors that can influence the family’s ability or capacity to provide emotional support for young people over the transition to adulthood. Factors with the most research (more than 20 articles) were: family proximity or co-residence; mental health; parenting styles; ethnicity and culture; sex or gender differences; socioeconomic status; family relationship quality; family communication; stage of development or age of the young person; and family structure. Factors with less research (fewer than 20 articles) were: societal context; young person’s romantic relationship status or transition to parenthood; young person’s sexual orientation or gender identity; personality; social networks; biological factors; physical health; adverse life events; and shared experiences, interests, and activities.

### Gaps in the Research Field and Suggestions for Future Research

Drawing on learning from the scoping review and the stakeholder consultation, we have identified the following gaps in existing research and suggestions for future research.

#### A Whole Household (and Beyond) Approach

The findings from our review reflect general trends observed within the wider literature on family relations over the transition to adulthood. In general, there has been considerably less focus on sibling relationships in existing research, as compared to the parent–child relationship where the bulk of the research lies (Conger and Little, 2010; Oliveira et al., 2020). Even less research in this context has focused on young people’s relationships with their grandparents and other extended family members (Lindell and Campione-Barr, 2017; Oliveira et al., 2020). Moreover, studies have often relied upon data collected from a single group of informants, such as young people or their parents or a selected sibling (Lindell and Campione-Barr, 2017; Oliveira et al., 2020).

Future research exploring the factors that influence the ability or capacity of the family to provide emotional support for young people over the transition to adulthood should focus on the whole family. In doing so, researchers should seek to collect data from multiple informants, including family members who live inside or outside of the household, and examine the factors influencing emotional support provision within the parent–child relationship as well as within relationships with siblings and extended family members. It is important for future research to consider and measure multiple family member perspectives, as family members may have different views on and experiences of their relationships with each other (Lindell and Campione-Barr, 2017; Oliveira et al., 2020).

We also identified minimal research seeking to compare similarities and differences in the factors influencing familial emotional support between families of varying size and structure, between family members with differing sexual orientations and gender identities, and between different types of families, including biological, married, cohabiting, separated, single-parent, step, adoptive, foster, and assisted conception families. Thus, future research should also seek to compare perceptions and experiences of familial emotional support, and the factors that influence this, within samples of participants from different family types and structures.

#### Contextually Situated Research

In another recent review of the literature, Fingerman et al. (2020) concluded that *“despite decades of head nodding to life course and ecological theories, many studies on intergenerational ties examine […] behaviors and qualities of this tie devoid of larger context”* (p. 394). Following Fingerman et al. (2020), we similarly conclude that more attention is needed in future research to the wider societal, political, cultural, and historical context within which family relationships sit, and the external factors (including government policies and shifts in the political and economic climate) that can influence the family’s ability or capacity to provide emotional support for young people over the transition to adulthood. Engagement with contextually situated meanings of mental health, wellbeing, family, and emotional support from participants’ perspectives, and within studies across different contexts, would also add to the research area.

#### Diversity in Samples

Studies have often recruited convenience samples (frequently students), with little heterogeneity in sex, ethnicity, and SES, thus limiting the generalisability of results. Males (e.g., young men and fathers) have been underrepresented across studies. The reliance on college student samples means that families of lower SES are likely underrepresented in existing literature. The majority of the research that we identified through our review was conducted in a United States context. Moreover, broad ethnic and cultural differentiations, such as between Western and non-Western societies, in existing research further limits the generalisability of findings, as there will likely be important subgroup differences within different geographic contexts and ethnic and cultural groups.

Consequently, echoing previous reviews of the literature on family relations over the transition to adulthood (Lindell and Campione-Barr, 2017; Oliveira et al., 2020), future research is needed to replicate and extend key results reported in our review with more diverse samples (e.g., families from a range of geographic locations, families of varying size and structure, and families of varying SES or class). In particular, given that the majority of the studies identified in our review were conducted in the United States, this suggests that there is a need for greater ethnic and cultural diversity in research in this area. This includes exploring familial emotional support provision and the factors that influence this in the context of migrant and majority cultures in different countries, indigenous communities, immigration status, being a refugee or asylum seeker, and experiences of racialization, racism, and discrimination. A range of possible factors influencing familial emotional support provision need to be explored in tandem by researchers when investigating differences between ethnic and cultural groups, including structural inequalities, discrimination, and the influence of national policies.

#### Longitudinal Research

The self-report and retrospective nature of much of the data identified through our review, and the cross-sectional and correlational design of many studies, limits conclusions that can be drawn about causal relations between factors and familial emotional support provision. Therefore, more longitudinal studies are needed to examine the factors that unfold over the transition to adulthood and influence the ability or capacity of the family to provide young people with emotional support.

#### Mixed Methods Research

Much of the research identified through our review is quantitative in design, with comparatively few studies taking a mixed methods or qualitative research approach. Research using both quantitative and qualitative methodologies could illuminate both the factors and mechanisms behind impact, i.e., *how* do factors exert their effect. Standardized measures of factors, emotional support, and outcomes can provide data to assess statistical change, patterns, or trends. However, some studies identified through our review have used very brief measures of emotional support, such as a single item. Future research should incorporate more complex measures of support, contact, and relationship quality when examining familial emotional support provision. In-depth qualitative research is then needed to explore the nature of emotional support provision by family members for young people, and the ways in which particular factors enable or hinder this, in participants’ own words and from their own lived experiences.

#### An Intersectional Approach

Consideration of the intersection between multiple aspects or facets of family members’ identities (including their sexual, gender, and ethnic identities) that could influence familial emotional support provision for young people is important and appears to be a gap in existing research. Future research should also consider how multiple factors intersect to either provide cumulative advantage or cumulative disadvantage for a family’s ability or capacity to provide emotional support for young people over the transition to adulthood.

### Limitations of This Review

The studies included in our scoping review represent those identified using our pre-defined search terms, which focused specifically on the transition to adulthood and familial emotional support provision. Given the exploratory nature of our review, emotional support was defined very broadly as a construct synonymous with multiple characteristics of familial support and relationships, which meant that our pool of eligible studies was large. Nonetheless, there is still an even broader literature relating to many of the factors identified here that would not have met the criteria for inclusion in our review based on our search terms. As we conducted a scoping review rather than a systematic review, we did not conduct a formal quality assessment of any of the studies included in this review.

Practical constraints placed several restrictions on our review process. First, only full texts that were available and accessible online were accessed during the review. Second, a systematic search of the gray literature was not conducted. Third, while all study titles and abstracts were double screened by two members of the review team, both full text screening and data extraction were conducted by single members of the review team. Double reviewer input at all stages of the review would have been desirable to maximize precision.

We excluded studies from our review if they focused on the experiences of young people and/or families with a physical health disorder, illness or disability, learning difficulty or disability, or neurodevelopmental disorder. Similarly, studies were also excluded from our review if they focused on evaluating the influence of a specific family support intervention. This meant that we focused to a lesser extent in our review on the family’s interaction with institutions, such as statutory care services, which could also affect familial emotional support provision. Given the large volume of research across both of these areas, there is much scope for future reviews focusing on the factors influencing emotional support provision within families with these experiences.

Given that the majority of the studies identified in our review were conducted with samples in the United States and other Western countries, the transferability of the factors identified through our review to other countries, cultures, and locations cannot be ascertained here. Our exclusion of studies not written in the English language may also have contributed to this. Moreover, our stakeholder interviewees were all based in the United Kingdom. We aimed to engage with a targeted yet diverse sample of stakeholders working within a range of sectors. Despite this, a limitation of the stakeholder consultation was the lack of representation of stakeholders from different sectors reflecting perspectives on different family structures (e.g., adoptive families and stepfamilies), different institutions (e.g., the criminal justice system), and different cultural perspectives. It is probable that a larger number and wider representation of stakeholders could have generated additional insights.

## Conclusion

Overall, our review suggests that future research examining the factors that enable or hinder the family in providing emotional support for young people over the transition to adulthood needs to take an approach that:

(1) Considers the intersectionality between the different factors influencing familial emotional support provision and between the different facets of family members’ and young people’s identities;
(2) Moves beyond focusing on the parent–child relationship to focusing on other family relationships too, including young people’s relationships with their siblings, grandparents, aunts and uncles, and cousins;
(3) Focuses on diverse samples in terms of sociodemographic characteristics, geographic locations, and family structures;
(4) Uses both quantitative and qualitative methodologies to illuminate the factors (and mechanisms behind the impact of factors) that influence familial emotional support provision over the transition to adulthood;
(5) Considers the wider context within which families are situated, including the influence of societal, political, economic, and cultural factors on the family’s ability or capacity to provide emotional support for young people;
(6) Seeks to apply current knowledge to the design, implementation, and evaluation of interventions or programs for supporting families in providing emotional support for young people over the transition to adulthood.

## Author Contributions

ES led on the conception and design of the study, literature searching and review process, stakeholder consultation, and overall drafting of the manuscript. IV contributed to the design of the study, project managed the literature searching and review process, and wrote sections of the manuscript. EL, MJ, and SF undertook the literature searching and review process, and wrote sections of the manuscript. HM contributed to the conception and design of the study, conducted the stakeholder interviews, and wrote sections of the manuscript. PC and MC contributed to the conception and design of the study, literature searching and review process, and wrote sections of the manuscript. RU contributed to the design of the study and drafting of the manuscript. All authors contributed to manuscript revision, read, and approved the submitted version.

## Author Disclaimer

The views expressed are those of the author(s) and not necessarily those of the Health Foundation.

## Conflict of Interest

RU is a partner in Riches & Ullman LLP. The remaining authors declare that the research was conducted in the absence of any commercial or financial relationships that could be construed as a potential conflict of interest.

## Publisher’s Note

All claims expressed in this article are solely those of the authors and do not necessarily represent those of their affiliated organizations, or those of the publisher, the editors and the reviewers. Any product that may be evaluated in this article, or claim that may be made by its manufacturer, is not guaranteed or endorsed by the publisher.
