# Supplementary material for: A Scoping Review of the Factors That Influence Families’ Ability or Capacity to Provide Young People With Emotional Support Over the Transition to Adulthood
Source: Front Psychol. 2021 Oct 14;12:732899. doi: 10.3389/fpsyg.2021.732899 (PMC8555465; doi:10.3389/fpsyg.2021.732899)
Supplement: Supplementary file 1 [file Table_2.docx]

| **Table 2.**Summary of key findings pertaining to factors affecting the family’s ability or capacity to provide emotional support for young people over the transition to adulthood | | | | |
| --- | --- | --- | --- | --- |
| **Factor** | **Studies** | **Country** | **Study design** | **Sample size** |
| **Adverse life events** | | | | |
|  | Allbaugh, 2018 | USA | Quantitative, Cross-sectional | Young people = 514 |
|  | Barnes et al., 2016 | USA | Quantitative, Cross-sectional | Young people = 304 |
|  | Benson &Bougakova, 2018 | USA | Mixed methods, Longitudinal | Young people = 450 |
|  | Berman et al., 2015 | USA | Qualitative, Cross-Sectional | Young people = 20 |
|  | Birgisdottir et al., 2019 | Sweden | Quantitative, Cross-sectional | Young people = 622;Matched peers = 330 |
|  | Duval et al., 2019 | Not specified | Quantitative, Cross-sectional | Young people = 793 |
|  | Fingerman et al., 2013b | USA | Quantitative, Cross-sectional | Parents =399; Young people =592 |
|  | Fingerman et al., 2020 | Worldwide –but with USA focus | Literature review | N/A |
|  | Gamache Martin, 2018 | USA | Quantitative, Cross-sectional | Young people and their mothers = 66 |
|  | McKinney et al., 2018 | USA | Quantitative, Cross-sectional | Young people = 2,362 |
|  | Murry, 2019 | USA | Quantitative, Longitudinal | Young people = 847 |
|  | Naughton et al., 2020 | Ireland | Quantitative, Cross-sectional | Young people = 465 |
|  | Nwokeji, 2009 | USA | Qualitative, Cross-sectional | Young people and their grandmothers = 18 |
|  | Nylander et al., 2018 | Sweden | Quantitative, Cross-sectional | Young people = 2,500 |
|  | Roach, 2019 | USA | Quantitative, Cross-sectional | Young people = 452 |
|  | Scott, 2010 | USA | Quantitative, Longitudinal | Young people and their fathers = 2,785 |
|  | Zhang & Flynn, 2020 | Australia | Qualitative, Cross-sectional | Young people = 5 |
| **Biological factors** | | | | |
|  | Elam et al., 2018 | USA | Qualitative, Longitudinal | Young people and their parents = 479 |
|  | Marceau et al., 2016 | USA | Quantitative, Cross-sectional | Families = 698 |
|  | Shirtcliff et al., 2017 | USA | Quantitative, Cross-sectional | Young people = 331 |
|  | Van der Cruijsen et al., 2019 | Netherlands | Quantitative, Cross-sectional | Young people = 160 |
| **Ethnicity and culture** | | | | |
|  | Alavi et al., 2020 | Worldwide | Quantitative, Cross-sectional | Young people = 600 |
|  | Atkin & Jackson, 2020 | USA | Qualitative, Cross-sectional | Young people = 20 |
|  | Baham, 2009 | USA | Quantitative, Cross-sectional | Young people = 322; Mothers and fathers = 322 |
|  | Bayly&Bumpus, 2020 | USA | Quantitative, Cross-sectional | Young people = 99; Mothers = 99 |
|  | Budescu & Silverman, 2016 | USA | Quantitative, Cross-sectional | Young people = 530 |
|  | Chang & Greenberger, 2012 | USA | Quantitative, Cross-sectional | Mothers = 140 |
|  | Chung et al., 2009 | USA | Quantitative, Longitudinal | Young people = 511 |
|  | Colaner et al., 2014 | USA | Quantitative, Cross-sectional | Young people = 409 |
|  | Cruz, 2007 | USA | Quantitative, Cross-sectional | Young people = 245 |
|  | Davis et al., 2018 | Columbia | Quantitative, Cross-sectional | Young people = 207 |
|  | Dillon et al., 2012 | USA | Quantitative, Cross-sectional | Young people = 527 |
|  | Edmond, 2008 | USA | Quantitative, Cross-sectional | Mothers = 415 |
|  | Fingerman et al., 2011 | USA | Quantitative, Cross-sectional | Young people and their parents = 658 |
|  | Fingerman et al., 2016c | Germany, Hong Kong, South Korea, USA | Quantitative, Cross-sectional | Young people = 1,301 |
|  | Fingerman, 2017 | Worldwide – but with USA focus | Literature review | N/A |
|  | Fingerman et al., 2020 | Worldwide – but with USA focus | Literature review | N/A |
|  | Guan & Shen, 2015 | USA | Quantitative, Cross-sectional | Young people = 139 |
|  | Guan &Fuligni, 2016 | USA | Quantitative, Longitudinal | Young people = 600 |
|  | Hardie & Seltzer, 2016 | USA | Quantitative, Cross-sectional | Young people = 6,962 |
|  | Hartnett et al., 2018 | USA | Quantitative, Cross-sectional | Young people = 5,090 |
|  | Hwang et al., 2019 | USA | Quantitative, Longitudinal | Mother–child dyads = 238; Father–child dyads = 148 |
|  | Karre, 2012 | USA | Quantitative, Cross-sectional | Young people = 414 |
|  | Khafi et al., 2014 | USA | Quantitative, Longitudinal | Young people = 361 |
|  | Killoren et al., 2014 | USA | Quantitative, Cross-sectional | Young people = 186 |
|  | Killoren et al., 2015 | USA | Quantitative, Cross-sectional | Families = 246 |
|  | Killoren et al., 2016 | USA | Quantitative, Cross-sectional | Young people = 186 |
|  | Killoren et al., 2017 | USA | Quantitative, Longitudinal | Families = 246 |
|  | Kim, 2008 | South Korea | Quantitative, Cross-sectional | Young people and their parents = 181 |
|  | Lanza et al., 2013 | USA | Quantitative, Cross-sectional | Young people = 4,743 |
|  | Lindell & Campione-Barr, 2017 | Worldwide | Literature review | N/A |
|  | Malkin & McKinney, 2019 | USA | Quantitative, Cross-sectional | Young people = 1,401 |
|  | Mansson et al., 2017 | USA | Quantitative, Cross-sectional | Young people = 171 |
|  | Mansson&Sigurdardottir, 2019 | Denmark, Iceland, Poland, USA | Quantitative, Cross-sectional | Young people = 606 |
|  | Maru, 2021 | USA | Quantitative, Longitudinal | Young people = 7,017 |
|  | Moilanen&Raffaelli, 2010 | USA | Quantitative, Cross-sectional | Young people = 495 |
|  | Morales et al., 2020 | USA | Qualitative | Young people = 9 |
|  | Munford & Sanders, 2016 | New Zealand | Qualitative, Cross-sectional | Young people (survey) = 593; Young people (interviews) = 107; Foster parents = 21 |
|  | Murry, 2019 | USA | Quantitative, Longitudinal | Families = 867 |
|  | Oliveira et al., 2020 | 14 different countries | Systematic review | N/A |
|  | Padilla et al., 2020 | USA | Quantitative, Longitudinal | Families = 246 |
|  | Park, 2019 | USA | Quantitative, Longitudinal | Young people = 1,650 |
|  | Portner& Riggs, 2016 | USA | Quantitative, Cross-sectional | Young people = 575 |
|  | Rasmi et al., 2017 | Canada | Qualitative, Cross-sectional | Young people = 12 |
|  | Reid & Finley, 2010 | USA | Quantitative, Cross-sectional | Young people = 202 |
|  | Reis et al., 2009 | USA | Quantitative, Cross-sectional | Young people = 87 |
|  | Scott, 2010 | USA | Quantitative, Longitudinal | Young people and their fathers = 2,785 |
|  | Sheu, 2019 | USA | Qualitative, Cross-sectional | Young people = 10 |
|  | Shilo&Savaya, 2012 | Israel | Quantitative, Cross-sectional | Young people = 461 |
|  | Stearns & McKinney, 2020 | USA | Quantitative, Cross-sectional | Young people = 486 |
|  | Streit et al., 2018 | USA | Quantitative, Cross-sectional | Young people = 196 |
|  | Tanaka, 2016 | USA | Quantitative, Longitudinal | Young people = 327; Mothers = 369; Fathers = 369 |
|  | Umaña-Taylor et al., 2012 | USA | Quantitative, Longitudinal | Families = 240 |
|  | Updegraff et al., 2018 | USA | Quantitative, Longitudinal | Young people = 191 |
|  | Wu et al., 2018 | USA | Qualitative, Cross-sectional | Young people = 48 |
|  | Yahirun, 2013 | Netherlands, Germany | Quantitative, Longitudinal | Young people = 15,197 |
|  | Yahirun, 2019 | USA | Quantitative, Longitudinal | Young people = 4,712 |
|  | Zhang et al., 2019 | USA | Quantitative, Longitudinal | Young people = 2,442 |
| **Family communication** | | | | |
|  | Ames-Sikora et al., 2017 | USA | Quantitative, Cross-sectional | Young people = 175 |
|  | Bacigalupe&Brauninger, 2017 | Spain | Qualitative | Young people = 12 |
|  | Brooks, 2015 | USA | Qualitative | Young people = 14 |
|  | Colaner et al., 2014 | USA | Quantitative, Cross-sectional | Young people = 409 |
|  | Conger & Little, 2010 | USA | Literature review | N/A |
|  | Deterding, 2010 | USA | Mixed Methods, Cross-sectional | Young people = 123 |
|  | DiTunnariello, 2016 | USA | Quantitative, Cross-sectional | Young people = 503 |
|  | Donovan et al., 2017 | USA | Quantitative, Cross-sectional | Young people = 298 |
|  | Fingerman et al., 2012a | USA | Literature review | Young people = 592; Parents = 633 |
|  | Fingerman et al., 2012b | USA | Quantitative, Cross-sectional | Young people = 592; Parents = 399 |
|  | Fingerman et al., 2012c | USA | Quantitative, Cross-sectional | Young people = 597; Parents = 381 |
|  | Fingerman et al., 2013a | USA | Literature review | N/A |
|  | Fingerman et al., 2016a | USA | Quantitative, Cross-sectional | Parents = 247; Young people = 578 |
|  | Fingerman et al., 2016c | Germany, Hong Kong, Korea, and the United States | Quantitative, Cross-sectional | Young people = 1,301 |
|  | Fingerman, 2017 | Worldwide – but with USA focus | Literature review | N/A |
|  | Fingerman et al., 2020 | Worldwide – but with USA focus | Literature review | N/A |
|  | Gamache Martin, 2018 | USA | Quantitative, Cross-sectional | Young people and their mothers = 66 |
|  | Gentzler et al., 2011 |  | Quantitative, Cross-sectional | Young people = 211 |
|  | Ghali, 2010 | USA | Quantitative, Longitudinal | Young people = 233 |
|  | Gungordu& Hernandez-Reif, 2020 | Not specified | Quantitative, Cross-sectional | Young people = 205 |
|  | Hessel & Dworkin, 2018 | USA | Systematic review | N/A |
|  | Jimenez, 2008 | Spain | Quantitative, Longitudinal | Young people = 101 |
|  | Kanter et al., 2012 | USA | Quantitative, Longitudinal | Parent–child dyads =18 |
|  | Lindell et al., 2015 | USA | Quantitative, Cross-sectional | Young people = 250 |
|  | Lindell & Campione-Barr, 2017 | Worldwide | Literature review | N/A |
|  | Luerssen et al., 2019 | USA | Quantitative, Cross-sectional | Young people = 802 |
|  | Marchant &O’Donohoe, 2014 | UK | Qualitative, Cross-sectional | Young people = 20 |
|  | Maximo & Carranza, 2016 | Philippines | Quantitative, Cross-sectional | Young people = 843 |
|  | Miller-Ott et al., 2014 | USA | Quantitative, Cross-sectional | Young people = 207 |
|  | O’Mara &Schrodt, 2017 | USA | Quantitative, Cross-sectional | Young people = 241 |
|  | Padilla-Walker & Daye, 2019 | USA | Quantitative, Longitudinal | Young people and their parents = 463 |
|  | Peters & Ehrenberg, 2008 | USA | Quantitative, Cross-sectional | Young people = 203 |
|  | Phillips &Schrodt, 2015a | USA | Quantitative, Cross-sectional | Young people = 329 |
|  | Roos et al., 2019 | South Africa | Qualitative | Young people = 18 |
|  | Saeed & Hanif, 2014 | Pakistan | Quantitative, Cross-sectional | Young people = 567 |
|  | Schrodt et al., 2007 | USA | Quantitative, Cross-sectional | Young people = 646 |
|  | Schrodt& Phillips, 2016 | USA | Quantitative, Cross-sectional | Young people = 329 |
|  | Schrodt& Afifi, 2018 | USA | Quantitative, Cross-sectional | Families = 170 |
|  | Scruggs & Paul, 2020 | USA | Quantitative, Cross-sectional | Young people = 235 |
|  | Segrin et al., 2012 | USA | Quantitative, Cross-sectional | Parent-child dyads = 538 |
|  | Sheu, 2019 | USA | Qualitative, Cross-sectional | Young people = 10 |
|  | Smout et al., 2020 | Australia | Quantitative, Cross-sectional | Young people = 442 |
|  | van Harmelen et al., 2016 | UK | Quantitative, Longitudinal | Young people = 771 |
|  | Whiteman et al., 2011 | USA | Quantitative, Longitudinal | Families = 184 |
|  | Yang, 2018 | USA | Qualitative, Cross-sectional | Young people = 51 |
|  | Yu et al., 2017 | China | Quantitative, Cross-sectional | Young people = 30 |
|  | Zuckerman, 2018 | USA | Quantitative, Cross-sectional | Young people = 50 |
| **Family proximity or co-residence** | | | | |
|  | Arenas Nieves, 2019 | USA | Qualitative, Cross-sectional | Young people = 15 |
|  | Bertogg&Szydlik, 2016 | Switzerland | Quantitative, Longitudinal | 4,306 dyads: Daughter-mother, son-mother, daughter-father, son-father |
|  | Brooks, 2015 | USA | Qualitative | Young people = 14 |
|  | Conger & Little, 2010 | USA | Literature review | N/A |
|  | Fabricius&Luecken, 2007 | USA | Quantitative, Cross-sectional | Young people = 266 |
|  | Feistman et al., 2016 | USA | Qualitative | Young people = 33 |
|  | Fingerman et al., 2016c | Germany, Hong Kong, South Korea, USA | Quantitative, Cross-sectional | Young people = 1,301 |
|  | Fingerman, 2017 | Worldwide - but with USA focus | Literature review | N/A |
|  | Fingerman et al., 2017 | USA | Quantitative, Cross-sectional | Young people = 159 |
|  | Gillespie &Treas, 2017 | USA | Quantitative, Longitudinal | Young people = 3,985 |
|  | Frank, 2007 | Canada | Quantitative, Cross-sectional | Young people = 207 |
|  | Galambos et al., 2018 | Canada | Quantitative, Longitudinal | Young people = 923 |
|  | Ganong et al., 2011 | USA | Qualitative, Cross-sectional | Young people = 49 |
|  | Hamwey et al., 2019 | USA | Qualitative | Young people = 454 |
|  | Kim et al., 2020 | USA | Quantitative, Cross-sectional | Parent–child dyads = 2,252 |
|  | Lindell & Campione-Barr, 2017 | Worldwide | Literature review | N/A |
|  | Milevsky &Heerwagen, 2013 | USA | Qualitative, Cross-sectional | Young people = 56 |
|  | Oliveira et al., 2020 | 14 different countries | Systematic review | N/A |
|  | Peters & Ehrenberg, 2008 | North America | Quantitative, Cross-sectional | Young people = 203 |
|  | Petree, 2014 | USA | Quantitative, Longitudinal | Young people = 3,135 |
|  | Roos et al., 2019 | South Africa | Mixed methods | Young people = 18 |
|  | Scott, 2010 | USA | Quantitative, Longitudinal | Young people and their fathers = 2,785 |
|  | Seiffge-Krenke, 2010 | Germany | Quantitative, Longitudinal | Young people = 93; Mothers = 93; Fathers = 68 |
|  | Sulimani-Aidan et al., 2017 | Israel | Quantitative, Cross-sectional | Young people = 231 |
|  | Whiteman et al., 2011 | USA | Quantitative, Longitudinal | Families = 184 |
|  | Wise &Onol, 2020 | Turkey | Quantitative, Cross-sectional | Young people = 208 |
|  | Zupancic et al., 2014 | Slovenia | Quantitative, Cross-sectional | Young people = 674 |
| **Family relationship quality** | | | | |
|  | Ahrons, 2007 | USA | Qualitative, Longitudinal | Young people = 173 |
|  | Alavi et al., 2020 | Worldwide | Quantitative, Cross-sectional | Young people = 600 |
|  | Bahrassa, 2013 | USA | Mixed methods, Longitudinal | Young people = 878 |
|  | Fabricius&Luecken, 2007 | USA | Quantitative, Cross-sectional | Young people = 266 |
|  | Fingerman et al., 2012a | USA | Literature review | Young people = 592; Parents = 633 |
|  | Fingerman et al., 2013b | USA | Quantitative, Cross-sectional | Parents = 399; Young people = 592 |
|  | Fingerman et al., 2016a | USA | Quantitative, Cross-sectional | Young people = 1,301 |
|  | Fingerman, 2017 | Worldwide – but with USA focus | Literature review | N/A |
|  | Frank, 2008 | Canada | Quantitative, Cross-sectional | Young people = 207 |
|  | Galambos et al., 2018 | Canada | Quantitative, Longitudinal | Young people = 923 |
|  | Ganong et al., 2011 | USA | Cross-sectional/qualitative | Young people = 49 |
|  | Ghali, 2010 | USA | Quantitative, Longitudinal | Young people = 233 |
|  | Gillespie &Treas, 2017 | USA | Quantitative, Longitudinal | Young people = 3,985 |
|  | Keijsers et al., 2011 | USA | Quantitative, Longitudinal | Young people = 503 |
|  | Kibblewhite, 2008 | Canada | Mixed methods, Cross-sectional | Young people = 30; Siblings = 30 |
|  | Levpuscek&Gril, 2010 | Slovenia | Quantitative, Cross-sectional | Young people = 546 |
|  | Lindell & Campione-Barr, 2017 | Worldwide | Literature review | N/A |
|  | O’Mara &Schrodt, 2017 | USA | Quantitative, Cross-sectional | Young people = 241 |
|  | Mansson et al., 2017 | USA | Quantitative, Cross-sectional | Young people = 171 |
|  | Maximo & Carranza, 2016 | Philippines | Quantitative, Cross-sectional | Young people = 843 |
|  | McKinney et al., 2018 | USA | Quantitative, Cross-sectional | Young people = 2,362 |
|  | Mendonca & Fontaine, 2013 | Portugal | Quantitative, Cross-sectional | Young people = 457 |
|  | Milevsky, 2019 | USA | Quantitative, Cross-sectional | Young people = 432 |
|  | Mizzi et al., 2020 | Australia | Quantitative, Cross-sectional | Young people = 70 |
|  | Monserud, 2008 | USA | Quantitative, Cross-sectional | Young people = 841 |
|  | Padilla et al., 2018 | USA | Quantitative, Longitudinal | Families = 246 |
|  | Padilla-Walker & Daye, 2019 | USA | Quantitative, Longitudinal | Young people and their parents = 463 |
|  | Pantelis et al., 2015 | Greece | Qualitative, Cross-sectional | Young people = 2 |
|  | Peters & Ehrenberg, 2008 | USA | Quantitative, Cross-sectional | Young people = 203 |
|  | Petrowski& Stein, 2016 | USA | Qualitative, Cross-sectional | Young people = 10 |
|  | Phillips &Schrodt, 2015b | USA | Quantitative, Cross-sectional | Young people = 329 |
|  | Ponti & Martina, 2019 | Italy | Quantitative, Cross-sectional | Young people = 253 |
|  | Preston et al., 2016 | USA | Quantitative, Cross-sectional | Young people = 130 |
|  | Riggio & Valenzuela, 2011 | USA | Quantitative, Cross-sectional | Young people = 431 |
|  | Roos et al., 2019 | South Africa | Mixed methods, Cross-sectional | Young people = 18 |
|  | Rowen & Emery, 2014 | USA | Quantitative, Cross-sectional | Young people = 677 |
|  | Rowen & Emery, 2019 | USA | Quantitative, Cross-sectional | Young people = 994 |
|  | Shanahan et al., 2008 | USA | Quantitative, Longitudinal | Families = 201 |
|  | Tibbetts &Scharfe, 2015 | Canada | Quantitative, Cross-sectional | Young people = 392 |
|  | Tucker et al., 2019 | USA | Quantitative, Longitudinal | Young people = 143 |
|  | Wise &Onol, 2020 | Turkey | Quantitative, Cross-sectional | Young people = 208 |
|  | Yu, 2007 | USA | Quantitative, Longitudinal | Young people = 30 |
|  | Zimmer-Gembeck et al., 2011 | USA | Quantitative, Cross-sectional | Young people = 206 |
| **Family structure** | | | | |
|  | Ahrons, 2007 | USA | Qualitative, Longitudinal | Young people = 173 |
|  | Baham, 2009 | USA | Quantitative, Cross-sectional | Young people = 322; Mothers and fathers = 322 |
|  | Benson &Bougakova, 2018 | USA | Mixed methods, Longitudinal | Young people = 450 |
|  | Bertogg&Szydlik, 2016 | Switzerland | Quantitative, Longitudinal | 4,306 dyads: Daughter-mother, son-mother, daughter-father, son-father |
|  | Cassidy et al., 2014 | UK | Quantitative, Cross-sectional | Young people = 708 |
|  | Coe, 2018 | USA | Quantitative, Cross-sectional | Young people = 313 |
|  | Conger & Little, 2010 | USA | Literature review | N/A |
|  | Fabricius&Luecken, 2007 | USA | Quantitative, Cross-sectional | Young people = 266 |
|  | Feistman et al., 2016 | USA | Qualitative | Young people = 33 |
|  | Ferrari et al., 2015 | Italy | Quantitative, Cross-sectional | Young people = 160 |
|  | Fingerman et al., 2009 | USA | Quantitative, Cross-sectional | Young people = 1,384; Parents = 633 |
|  | Fingerman et al., 2012a | USA | Quantitative, Cross-sectional | Young people = 592; Parents = 399 |
|  | Fingerman et al., 2015 | USA | Quantitative, Cross-sectional | Young people = 1,384; Parents = 633 |
|  | Fingerman, 2017 | Worldwide – but with USA focus | Literature review | N/A |
|  | Fingerman et al., 2020 | Worldwide – but with USA focus | Literature review | N/A |
|  | Finzi-Dottan& Cohen, 2011 | Israel | Quantitative, Cross-sectional | Young people = 202 |
|  | Frank, 2007 | Canada | Quantitative, Cross-sectional | Young people = 207 |
|  | Frank, 2008 | Canada | Quantitative, Cross-sectional | Young people = 207 |
|  | Ganong et al., 2011 | USA | Qualitative, Cross-sectional | Young people = 49 |
|  | Goldberg, 2015 | USA | Quantitative, Longitudinal | Young people = 4,887 |
|  | Golombok et al., 2009 | UK | Quantitative, Longitudinal | Young people = 109 |
|  | Gillespie &Treas, 2017 | USA | Quantitative, Longitudinal | Young people = 3,985 |
|  | Hair et al., 2008 | USA | Quantitative, Longitudinal | Young people = 4,671 |
|  | Hartnett et al., 2018 | USA | Quantitative, Cross-sectional | Young people = 5,090 |
|  | Hoenayi&Yendork, 2018 | Ghana | Qualitative, Cross-sectional | Young people = 16 |
|  | Jensen &Lippold, 2018 | USA | Quantitative, Longitudinal | Young people = 20,745 |
|  | Kim et al., 2020 | USA | Quantitative, Cross-sectional | Parent–child dyads = 2,252 |
|  | King & Lindstrom, 2016 | USA | Quantitative, Longitudinal | Parents (usually mother) = 17,670; Young people (Wave I) = 20,745 |
|  | Lindell & Campione-Barr, 2017 | Worldwide | Literature review | N/A |
|  | Milevsky, 2019 | USA | Quantitative, Cross-sectional | Young people = 432 |
|  | Milevsky &Heerwagen, 2013 | USA | Qualitative, Cross-sectional | Young people = 56 |
|  | Miller, 2010 | Canada | Qualitative, Cross-sectional | Young people = 9 |
|  | Munford & Sanders, 2016 | New Zealand | Qualitative, Cross-sectional | Young people (survey) = 593; Young people (interviews) = 107; Foster parents = 21 |
|  | Nicholson, 2007 | USA | Quantitative, Cross-sectional | Young people = 31 |
|  | Nitzburg, 2013 | USA | Quantitative, Cross-sectional | Young people = 1,167 |
|  | Oliveira et al., 2020 | 14 different countries | Systematic review | N/A |
|  | O’Mara &Schrodt, 2017 | USA | Quantitative, Cross-sectional | Young people = 241 |
|  | Owen &Golombok, 2009 | UK | Quantitative, Longitudinal | Parents (mothers and fathers) = 90 |
|  | Padilla et al., 2018 | USA | Quantitative, Longitudinal | Families = 246 |
|  | Pantelis et al., 2015 | Greece | Qualitative, Cross-sectional | Young people = 2 |
|  | Peters & Ehrenberg, 2008 | USA | Quantitative, Cross-sectional | Young people = 203 |
|  | Reed et al., 2016 | USA | Qualitative, Cross-sectional | Young people = 15 |
|  | Riggio & Valenzuela, 2011 | USA | Quantitative, Cross-sectional | Young people = 431 |
|  | Rowen & Emery, 2014 | USA | Quantitative, Cross-sectional | Young people = 677 |
|  | Rowen & Emery, 2019 | USA | Quantitative, Cross-sectional | Young people = 994 |
|  | Schaan et al., 2019 | Germany | Quantitative, Cross-sectional | Young people = 121 |
|  | Scott, 2010 | USA | Quantitative, Longitudinal | Young people and their fathers = 2,785 |
|  | Soliz, 2007 | USA | Quantitative, Cross-sectional | Young people = 88 |
|  | Sulimani-Aidan, 2019 | Israel | Qualitative, Cross-sectional | Young people = 32 |
|  | Walkner&Rueter, 2014 | USA | Quantitative, Longitudinal | Young people = 473; Mothers = 470; Fathers = 418 |
|  | Walkner-Spaan, 2016 | USA | Quantitative, Longitudinal | Young people (self-report) = 272; Young people (observation) = 273 |
|  | Ward et al., 2019 | USA | Quantitative, Cross-sectional | Young people = 226 |
|  | Won, 2009 | USA | Quantitative, Longitudinal | Young people = 484 |
|  | Wu et al., 2018 | USA | Qualitative, Cross-sectional | Young people = 48 |
|  | Young & Ehrenberg, 2007 | Canada | Quantitative, Cross-sectional | Young people = 368 |
|  | Yu, 2007 | USA | Quantitative, Longitudinal | Young people = 30 |
| **Mental health** | | | | |
|  | Alhussain et al., 2019 | USA | Mixed methods | Young people = 137 |
|  | Boutelle et al., 2009 | USA | Quantitative, Longitudinal | Young people = 2,516 |
|  | Brawer, 2018 | USA | Qualitative, Cross-sectional | Young people = 15 |
|  | Brook et al., 2015 | USA | Quantitative, Longitudinal | Parent-child dyads = 281 |
|  | Chung et al, 2009 | USA | Quantitative, Longitudinal | Young people = 511 |
|  | Franz & McKinney, 2018 | USA | Quantitative, Cross-sectional | Young people = 665 |
|  | Gamache Martin, 2018 | USA | Quantitative, Cross-sectional | Young people and their mothers = 66 |
|  | Jacoby & Heatherington, 2016 | USA | Quantitative, Cross-sectional | Young people = 64 |
|  | Katz et al., 2013 | USA | Quantitative, Longitudinal | Young people = 182 |
|  | Kim, 2012 | USA | Quantitative, Longitudinal | Young people = 775 |
|  | Li et al., 2019 | International | A meta-analysis (a variety of methods) | Young people = 164,459 |
|  | McKinney &Milone, 2012 | USA | Quantitative, Cross-sectional | Young people = 328 |
|  | McKinney et al., 2018 | USA | Quantitative, Cross-sectional | Young people = 2,362 |
|  | Mitchell & Abraham, 2018 | USA | Mixed methods, Cross-sectional | Young people = 196 |
|  | Moberg, 2011 | Swedish | Quantitative, Longitudinal | Young people = 2,369 (Wave I), 1,705 (Wave II) |
|  | Needham, 2008 | USA | Quantitative, Longitudinal | Young people = 10,828 |
|  | Padilla et al., 2018 | USA | Quantitative, Longitudinal | Families = 246 |
|  | Petrowski& Stein, 2016 | USA | Qualitative, Cross-sectional | Young people = 10 |
|  | Roche et al., 2016 | Ghana | Quantitative, Longitudinal | Young people = 718 |
|  | Scott, 2010 | USA | Quantitative, Longitudinal | Young people and their fathers = 2,785 |
|  | Stormshak et al., 2019 | USA | Quantitative, Longitudinal | Young people and their parents = 593 |
|  | Sulimani-Aidan, 2019 | Israel | Quantitative, Cross-sectional | Young people = 32 |
|  | Szkody& McKinney, 2020 | USA | Quantitative, Cross-sectional | Young people = 430 |
|  | Verbeke et al., 2017 | Denmark | Quantitative, Cross-sectional | Young people = 205 |
|  | Ward et al., 2019 | USA | Quantitative, Cross-sectional | Young people = 226 |
|  | Watson, 2019 | USA | Quantitative, Cross-sectional | Young people = 28 |
| **Parenting styles** | | | | |
|  | Clark Culpepper, 2007 | USA | Quantitative, Cross-sectional | Young people = 87 |
|  | Fingerman et al., 2012b | USA | Quantitative, Cross-sectional | Young people = 592; Parents = 399 |
|  | Fingerman et al., 2013a | USA | Literature review | N/A |
|  | Fingerman et al., 2013b | USA | Quantitative, Cross-sectional | Parents = 399; Young people = 592 |
|  | Finzi-Dottan& Cohen, 2011 | Israel | Quantitative, Cross-sectional | Young people = 202 |
|  | Gozu, 2017 | USA | Quantitative, Cross-sectional | Young people = 775 |
|  | Hamwey& Whiteman, 2020 | Not reported | Quantitative, Cross-sectional | Young people = 454 |
|  | Jensen, 2012 | USA | Quantitative, Cross-sectional | Sibling pairs = 151 |
|  | Jensen, 2013 | USA | Quantitative, Cross-sectional | Sibling pairs = 151 |
|  | Jiang et al., 2017 | Hong Kong | Quantitative, Cross-sectional | Young people = 490 |
|  | Jung et al., 2020 | USA and South Korea | Quantitative, Cross-sectional | Young people = 386 |
|  | Killoren et al., 2015 | USA | Quantitative, Cross-sectional | Families = 246 |
|  | Kim, 2008 | South Korea | Quantitative, Cross-sectional | Young people and their parents = 181 |
|  | Lindell & Campione-Barr, 2017 | Worldwide | Literature review | N/A |
|  | Milevsky, 2020 | USA | Quantitative, Longitudinal | Young people = 272 |
|  | Nelson et al., 2011 | USA | Quantitative, Cross-sectional | Young people = 403; Mothers = 317; Fathers = 287 |
|  | Padilla et al., 2018 | 2007  USA | Quantitative, Longitudinal | Families = 246 |
|  | Padilla-Walker et al., 2014 | USA | Quantitative, Cross-sectional | Young people = 438; Mothers = 376; Fathers = 303 |
|  | Phillips &Schrodt, 2015a | USA | Quantitative, Cross-sectional | Young people = 329 |
|  | Phillips &Schrodt, 2015b | USA | Quantitative, Cross-sectional | Young people = 329 |
|  | Portner& Riggs, 2016 | USA | Quantitative, Cross-sectional | Young people = 575 |
|  | Rote et al., 2020 | USA | Quantitative, Cross-sectional | Young people = 282 |
|  | Roth et al., 2016 | Israel | Quantitative, Cross-sectional | Study 1: Young people = 125; Study 2: Young people and their mothers = 128 |
|  | Shenaar-Golan & Goldberg, 2019 | Israel | Quantitative, Cross-sectional | Young people = 198 |
|  | Siennick, 2013 | USA | Quantitative, Longitudinal | Sibling pairs = 1,470 |
|  | Woo, 2020 | USA | Quantitative, Cross-sectional | Young people = 191 |
|  | Young & Ehrenberg, 2007 | Canada | Quantitative, Cross-sectional | Young people = 368 |
|  | Young, 2013 | Canada | Quantitative, Cross-sectional | Young people = 275 |
| **Personality** | | | | |
|  | Gozu, 2017 | USA | Quantitative, Cross-sectional | Young people = 775 |
|  | Lanthier, 2007 | USA | Quantitative, Cross-sectional | Sibling pairs = 123 |
|  | Neyer&Lehnart, 2007 | Germany | Quantitative, Longitudinal | Young people = 339 |
|  | Roos et al., 2019 | South Africa | Mixed methods, Cross-sectional | Young people = 18 |
|  | Walecka-Matyja, 2018 | Poland | Quantitative, Cross-sectional | Young people = 160 |
|  | Zupancic&Kavcic, 2014 | Slovenia | Quantitative, Cross-sectional | Young people = 674 |
| **Physical health** | | | | |
|  | Bertogg&Szydlik, 2016 | Switzerland | Quantitative, Longitudinal | 4,306 dyads: Daughter-mother, son-mother, daughter-father, son-father |
|  | Kim et al., 2020 | USA | Quantitative, Cross-sectional | Parent–child dyads = 2,252 |
|  | Roos et al., 2019 | South Africa | Mixed methods, Cross-sectional | Young people = 18 |
| **Sex or gender differences** | | | | |
|  | Agueda et al., 2015 | Spain | Quantitative, Longitudinal | Young people = 90 |
|  | Bertogg&Szydlik, 2016 | Switzerland | Quantitative, Longitudinal | 4,306 dyads: Daughter-mother, son-mother, daughter-father, son-father |
|  | Beyers &Goossens, 2008 | Belgium | Quantitative, Longitudinal | Young people = 639 |
|  | Bowles & Hattie, 2015 | Australia | Quantitative, Longitudinal | Young people = 137 |
|  | Cassidy et al., 2014 | UK | Quantitative, Cross-sectional | Young people = 708 |
|  | Fellers &Schrodt, 2020 | USA | Quantitative, Cross-sectional | Young people = 227 |
|  | Fingerman et al., 2012b | USA | Quantitative, Cross-sectional | Young people = 592 |
|  | Fingerman et al., 2016c | Germany, Hong Kong, Korea, and the United States | Quantitative, Cross-sectional | Young people = 1,301 |
|  | Fingerman, 2017 | Worldwide - but with USA focus | Literature review | N/A |
|  | Finzi-Dottan& Cohen, 2011 | Israel | Quantitative, Cross-sectional | Young people = 202 |
|  | Frank, 2007 | Canada | Quantitative, Cross-sectional | Young people = 207 |
|  | Franz & McKinney, 2018 | USA | Quantitative, Cross-sectional | Young people = 665 |
|  | Freeman& Almond, 2010 | USA | Mixed-methods, Cross-sectional | Young people = 1,012 |
|  | Galambos et al., 2018 | Canada | Quantitative, Longitudinal | Young people = 923 |
|  | Gray et al., 2019 | India | Mixed-methods, Cross-sectional | Young people = 377 |
|  | Gungordu& Hernandez-Reif, 2020 | USA | Quantitative, Cross-sectional | Young people = 205 |
|  | Hartnett et al., 2018 | USA | Quantitative, Cross-sectional | Young people = 5,090 |
|  | Jimenez, 2008 | Spain | Quantitative, Longitudinal | Young people = 101 |
|  | Kim, 2012 | USA | Quantitative, Longitudinal | Young people = 110 |
|  | Kim et al., 2020 | USA | Quantitative, Cross-sectional | Parent–child dyads = 2,252 |
|  | Lanza et al., 2013 | USA | Quantitative, Cross-sectional | Young people = 4,743 |
|  | Levitt et al., 2007 | USA | Quantitative, Longitudinal | Young people = 756 |
|  | Lindell & Campione-Barr, 2017 | Worldwide | Literature review | N/A |
|  | Lindell et al., 2020 | USA | Quantitative, Longitudinal | Young people = 260 |
|  | Maximo & Carranza, 2016 | Philippines | Quantitative, Cross-sectional | Young people = 843 |
|  | McKinney et al., 2018 | USA | Quantitative, Cross-sectional | Young people = 2,362 |
|  | Mendonca & Fontaine, 2013 | Portugal | Quantitative, Cross-sectional | Young people = 457 |
|  | Milevsky &Heerwagen, 2013 | USA | Qualitative, Cross-sectional | Young people = 52 |
|  | Miller, 2010 | Canada | Qualitative, Cross-sectional | Young people = 9 |
|  | Moilanen&Raffaelli, 2010 | USA | Quantitative, Cross-sectional | Young people = 495 |
|  | Monserud, 2008 | USA | Quantitative, Cross-sectional | Young people = 841 |
|  | Nelson et al., 2011 | USA | Quantitative, Cross-sectional | Young people = 403; Mothers = 317; Fathers = 287 |
|  | Neves et al., 2019 | Portugal | Mixed-methods, Longitudinal | Surveys (*N* = 1,650 young people); Interviews (*N* = 70 young people) |
|  | Oliveira et al., 2020 | 14 different countries | Systematic review | N/A |
|  | O'Mara &Schrodt, 2017 | USA | Quantitative, Cross-sectional | Young people = 241 |
|  | Padilla et al., 2018 | USA | Quantitative, Longitudinal | Families = 246 |
|  | Parra et al., 2015 | Spain | Quantitative, Longitudinal | Young people = 90 |
|  | Peters & Ehrenberg, 2008 | USA | Quantitative, Cross-sectional | Young people = 203 |
|  | Proulx & Helms, 2008 | USA | Mixed-methods, Cross-sectional | Parent-child dyads = 142 |
|  | Rossetto et al., 2017 | USA | Qualitative, Cross-sectional | Young people = 37 |
|  | Seo, 2007 | USA | Quantitative, Longitudinal | 362 households |
|  | Shilo&Savaya, 2012 | Israel | Quantitative, Cross-sectional | Young people = 461 |
|  | Suh &Fabricius, 2019 | USA | Quantitative, Longitudinal | Young people = 392 (Wave I), Young people = 287 (Wave II) |
|  | Sun-A et al., 2019 | USA | Quantitative, Longitudinal | Young people = 674 |
|  | Tibbetts &Scharfe, 2015 | Canada | Quantitative, Cross-sectional | Young people = 392 |
|  | Umaña-Taylor et al., 2012 | USA | Quantitative, Longitudinal | Families = 240 |
|  | Walecka-Matyja, 2018 | Poland | Quantitative, Cross-sectional | Young people = 160 |
|  | Wise, 2008 | USA | Quantitative, Cross-sectional | Young people = 208 |
|  | Zupancic et al., 2012 | Slovenia | Quantitative, Cross-sectional | Young people = 201 |
|  | Zupancic&Kavcic, 2014 | Slovenia | Quantitative, Cross-sectional | Young people = 674 |
| **Shared experiences, interests, and activities** | | | | |
|  | Bayly&Bumpus, 2020 | USA | Quantitative, Cross-sectional | Young people = 99; Mothers = 99 |
|  | Boer & Abubakar, 2014 | Kenya, the Philippines, New Zealand, and Germany | Quantitative, Cross-sectional | Young people = 760 |
|  | Colaner et al., 2014 | USA | Quantitative, Cross-sectional | Young people = 409 |
|  | Doughty, 2017 | USA | Quantitative, Longitudinal | Families (mothers, fathers, and young people) = 203 |
|  | Ganong et al., 2011 | USA | Qualitative, Cross-sectional | Young people = 49 |
|  | Hair et al., 2008 | USA | Quantitative, Longitudinal | Young people = 4,671 |
|  | Hammons, 2010 | USA | Quantitative, Cross-sectional | Young people = 485 |
|  | Hamwey et al., 2019 | USA | Qualitative, Cross-sectional | Young people = 454 |
|  | Hancock, 2013 | Not specified | Quantitative, Cross-sectional | Young people = 196 |
|  | Layland et al., 2020 | USA | Qualitative, Cross-sectional | Young people = 185 |
|  | Maximo & Carranza, 2016 | Philippines | Quantitative, Cross-sectional | Young people = 843 |
|  | Ponti & Martina, 2020 | Italy | Quantitative, Cross-sectional | Young people = 145 |
|  | Radmacher&Azmitia, 2016 | USA | Quantitative, Longitudinal | Young people = 82 |
|  | Wallace & Harwood, 2018 | USA | Quantitative, Cross-sectional | Young people = 173 |
| **Social networks** | | | | |
|  | Munford & Sanders, 2016 | New Zealand | Qualitative, Cross-sectional | Young people (survey) = 593; Young people (interviews) = 107; Foster parents = 21 |
|  | Reis et al., 2009 | USA | Quantitative, Cross-sectional | Young people = 187 |
| **Societal context** | | | | |
|  | Fingerman et al., 2012a | USA | Quantitative, Cross-sectional | Young people = 592; Parents = 399 |
|  | Fingerman et al., 2013a | USA | Literature review | N/A |
|  | Fingerman et al., 2016a | USA | Quantitative, Cross-sectional | Young people = 1,301 |
|  | Fingerman, 2017 | Worldwide – but with USA focus | Literature review | N/A |
|  | Fingerman et al., 2020 | Worldwide – but with USA focus | Literature review | N/A |
|  | Munford & Sanders, 2016 | New Zealand | Qualitative, Cross-sectional | Young people (survey) = 593; Young people (interviews) = 107; Foster parents = 21 |
|  | Oliveira et al., 2020 | Worldwide | Systematic review | N/A |
|  | Yahirun, 2013 | Netherlands and Germany | Longitudinal | Young people = 15,197 |
| **Socioeconomic status** | | | | |
|  | Bertogg&Szydlik, 2016 | Switzerland | Quantitative, Longitudinal | 4,306 dyads: Daughter-mother, son-mother, daughter-father, son-father |
|  | Brooks, 2015 | USA | Qualitative, Cross-Sectional | Young people = 14 |
|  | Conger & Little, 2010 | USA | Literature review | N/A |
|  | Fingerman et al., 2009 | USA | Quantitative, Cross-sectional | Young people = 1,384; Parents = 633 |
|  | Fingerman et al., 2012c | USA | Quantitative, Cross-sectional | Young people = 597; Parents = 381 |
|  | Fingerman et al., 2015 | USA | Quantitative, Cross-sectional | Parents = 633 and their children over age 18 = 1,384 |
|  | Fingerman, 2017 | Worldwide - but with USA focus | Literature review | N/A |
|  | Hartnett et al., 2018 | USA | Quantitative, Cross-sectional | Young people = 5,090 |
|  | Kim et al., 2020 | USA | Quantitative, Cross-sectional | Parent–child dyads = 2,252 |
|  | Levitt et al., 2007 | USA | Quantitative, Longitudinal | Young people = 756 |
|  | Johnson, 2013 | USA | Quantitative, Longitudinal | Young people = 9,128 to 13,389 across outcomes |
|  | Lim, 2012 | USA | Quantitative, Cross-sectional | Young people = 409 |
|  | Lindell, 2019 | USA | Quantitative, Longitudinal | Young people = 260 |
|  | Lindell et al., 2020 | USA | Quantitative, Longitudinal | Young people = 260 |
|  | Melby et al., 2008 | USA | Quantitative, Longitudinal | Young people = 451 |
|  | Murry, 2019 | USA | Quantitative, Longitudinal | Families = 867 |
|  | Neves et al., 2019 | Portugal | Mixed methods, Longitudinal | Surveys (*N* = 1,650 young people); Interviews (*N* = 70 young people) |
|  | Park, 2019 | USA | Quantitative, Longitudinal | Young people = 20,745 (Wave I), 15,701 (Wave IV) |
|  | Peters & Ehrenberg, 2008 | USA | Quantitative, Cross-sectional | Young people = 203 |
|  | Quan, 2020 | China | Quantitative, Cross-sectional | Young people = 203 |
|  | Roksa, 2019 | USA | Quantitative, Cross-sectional | Young people = 740 |
|  | Roy et al., 2010 | USA | Quantitative, Longitudinal | Young people = 297 |
|  | Scott, 2010 | USA | Quantitative, Longitudinal | Young people and their fathers = 2,785 |
|  | Umaña-Taylor et al., 2012 | USA | Quantitative, Longitudinal | Families = 240 |
|  | Zhang et al., 2019 | USA | Quantitative, Longitudinal | Young people = 2,442 |
| **Stage of development or age of the young person** | | | | |
|  | Agueda et al., 2015 | Spain | Quantitative, Longitudinal | Young people = 90 |
|  | Ana et al., 2020 | Portugal | Quantitative, Cross-sectional | Young people = 387 |
|  | Bowles & Hattie, 2015 | Australia | Quantitative, Longitudinal | Young people = 137 |
|  | Bucx& van Wel, 2008 | The Netherlands | Quantitative, Longitudinal | Young people = 1,064 |
|  | Conger & Little, 2010 | USA | Literature review | N/A |
|  | Fingerman et al., 2011 | USA | Quantitative, Cross-sectional | Parents = 397 |
|  | Fingerman et al., 2012b | USA | Quantitative, Cross-sectional | Young people = 592; Parents = 399 |
|  | Fingerman et al., 2013b | USA | Quantitative, Cross-sectional | Parents = 399; Young people = 592 |
|  | Fingerman et al., 2015 | USA | Quantitative, Cross-sectional | Parents = 633 and their young adult children = 1,384 |
|  | Fingerman et al., 2016c | Germany, Hong Kong, Korea, and the United States | Quantitative, Cross-sectional | Young people = 1,301 |
|  | Galambos et al., 2018 | Canada | Quantitative, Longitudinal | Young people = 923 |
|  | De Goede et al., 2009 | The Netherlands | Quantitative, Longitudinal | Young people = 1,341 |
|  | Guan &Fuligni, 2015 | USA | Qualitative, Longitudinal | Young people = 600 |
|  | Halliwell, 2016 | Not specified | Qualitative, Cross-sectional | Young people = 19 |
|  | Jensen et al., 2018 | Not specified | Quantitative, Longitudinal | Families = 180; Young people = 273 |
|  | Jimenez, 2008 | Spain | Mixed methods, Longitudinal | Young people = 101 |
|  | Jou, 2015 | USA | Quantitative, Longitudinal | Young people = 287 |
|  | Killoren et al., 2016 | USA | Quantitative, Cross-sectional | Young people = 186 |
|  | Lamborn &Moua, 2008 | USA | Qualitative, Cross-sectional | Young people = 40 |
|  | Lanz& Tagliabue, 2014 | Italy | Quantitative, Longitudinal | Parents and young people = 98 |
|  | Lindell et al., 2014 | USA | Quantitative, Longitudinal | Sibling pairs: 48 |
|  | Lindell & Campione-Barr, 2017 | Worldwide | Literature review | N/A |
|  | Lindell, 2019 | USA | Qualitative, Longitudinal | Young people = 260 |
|  | MaloneBeach et al., 2018 | USA | Quantitative, Cross-sectional | Young people = 470 |
|  | Melby et al., 2008 | USA | Quantitative, Longitudinal | Young people = 451 |
|  | Moilanen&Raffaelli, 2010 | USA | Quantitative, Cross-sectional | Young people = 495 |
|  | Morgan et al., 2010 | USA | Quantitative, Longitudinal | Young people = 30 |
|  | Nelson et al., 2011 | USA | Quantitative, Cross-sectional | Young people = 403; Mothers = 317; Fathers = 287 |
|  | Neves et al., 2019 | Portugal | Mixed methods, Longitudinal | Surveys (*N* = 1,650 young people); Interviews (*N* = 70 young people) |
|  | Neyer&Lehnart, 2007 | Germany | Quantitative, Longitudinal | Young people = 339 |
|  | Oliveira et al., 2020 | Worldwide | Systematic review | N/A |
|  | Padilla et al., 2018 | USA | Quantitative, Longitudinal | Families = 246 |
|  | Parra et al., 2015 | Spain | Quantitative, Longitudinal | Young people = 90 |
|  | Proulx & Helms, 2008 | USA | Mixed-methods, Cross-sectional | Parent-child dyads = 142 |
|  | Roche et al., 2016 | Ghana | Quantitative, Longitudinal | Young people = 718 |
|  | Rossetto et al., 2017 | USA | Qualitative, Cross-sectional | Young people = 37 |
|  | Scott, 2010 | USA | Quantitative, Longitudinal | Young people and their fathers = 2,785 |
|  | Spitz et al., 2020 | Switzerland | Quantitative, Longitudinal | Young people = 857 |
|  | Suh &Fabricius, 2019 | USA | Quantitative, Longitudinal | Young people = 392 (Wave I); Young people = 287 (Wave II) |
|  | Sulimani-Aidan, 2020 | Israel | Qualitative, Cross-sectional | Young people = 23 |
|  | Szwedo et al., 2017 | USA | Quantitative, Longitudinal | Young people = 184 |
|  | Tsai et al., 2013 | USA | Quantitative, Longitudinal | Young people = 821 |
|  | Wang, 2019 | Singapore | Quantitative, Cross-sectional | Parent–child dyads = 156 |
|  | Wetzel & Hank, 2020 | Germany | Quantitative, Longitudinal | Grandparents = 349; Grandchildren = 494 |
|  | Young & Ehrenberg, 2007 | Canada | Quantitative, Cross-sectional | Young people = 368 |
|  | Zupancic et al., 2014 | Slovene | Quantitative, Cross-sectional | Young people = 674 |
| **Young person’s romantic relationship status or transition to parenthood** | | | | |
|  | Fingerman et al., 2016b | USA | Quantitative, Cross-sectional | Young people = 1,301 |
|  | Fingerman et al., 2020 | Worldwide – but with USA focus | Literature review | N/A |
|  | Gillespie &Treas, 2017 | USA | Quantitative, Longitudinal | Young people = 3,985 |
|  | Kim et al., 2020 | USA | Quantitative, Cross-sectional | Parent–child dyads = 2,252 |
|  | Lanz&Tagliatue, 2007 | Italy | Quantitative, Cross-sectional | Young people = 176 |
|  | Shenhav, 2018 | USA | Quantitative, Cross-sectional | Young people = 588 |
|  | Suh &Fabricius, 2019 | USA | Quantitative, Longitudinal | Young people = 392 (Wave I); Young people = 287 (Wave II) |
|  | Updegraff et al., 2018 | USA | Quantitative, Longitudinal | Young people = 191 |
|  | Yahirun, 2019 | USA | Quantitative, Longitudinal | Young people = 4,712 |
| **Young person’s sexual orientation or gender identity** | | | | |
|  | Fingerman et al., 2020 | Worldwide – but with USA focus | Literature review | N/A |
|  | Lai et al., 2010 | Taiwan | Quantitative, Cross-sectional | Young people = 5,010 |
|  | Littman, 2018 | USA | Qualitative, Cross-sectional | Young people = 256 |
|  | Needham & Austin, 2010 | USA | Quantitative, Cross-sectional | Young people = 11,153 |
|  | Platt et al., 2020 | USA | Quantitative, Cross-sectional | Young people = 550 |
|  | Rosario et al., 2014a | USA | Quantitative, Longitudinal | Young people = 5,647 |
|  | Rosario et al., 2014b | USA | Quantitative, Longitudinal | Young people = 6,122 |
|  | Shilo&Savaya, 2012 | Israel | Quantitative, Cross-sectional | Young people = 461 |
