# Supplementary material for: A Scoping Review of the Factors That Influence Families’ Ability or Capacity to Provide Young People With Emotional Support Over the Transition to Adulthood
Source: Front Psychol. 2021 Oct 14;12:732899. doi: 10.3389/fpsyg.2021.732899 (PMC8555465; doi:10.3389/fpsyg.2021.732899)
Supplement: Supplementary file 2 [file Data_Sheet_1.docx]

**Supplementary Materials**

**Appendix A: Scoping review search terms**

(adulthood transition or early adult* or emerging adult* or transition to adult* or young adult* or late adolescen* or older adolescen* or ((transition* or prepar* or readiness or ready) adj3 adult*))

AND

(family or families or familial or father* or grandfather* or grandmother* or grandparent* or intergenerational or kinship or maternal or mother* or parent* or paternal or stepfamil* or step famil* or stepfather* or step father* or stepmother* or step mother* or sibling*)

AND

(affection* or cohesive family or emotional* availab* or emotional bond or emotional* close* or emotional communication or emotional engagement or emotional* expressi* or emotional* open* or emotional support or empath* or family cohesion or family connectedness or family dynamics or family relations* or family expressiveness or famil* support or father-child communication or father-child interaction* or father-child relations* or maternal acceptance or maternal expressiveness or maternal support* or maternal responsiveness or maternal sensitivity or mother-child communication or mother-child interaction* or mother-child relations* or moral support or parent-child communication or parent child communication or parent-child interaction* or parent child interaction* or parent-child relations* or parent child relations* or parental acceptance or parental expressiveness or parental responsiveness or parental sensitivity or parental support* or paternal expressiveness or paternal responsiveness or paternal acceptance or paternal sensitivity or paternal support* or sibling support* or sibling relations* or social support or supportive father* or supportive mother* or supportive parent* or supportive sibling* or supportive famil* or warm*)

**Appendix B: Stakeholder interview schedule**

1. Could we start with you describing what your role is at [organisation]?
2. Can you tell me about your work with young people and/or families?
3. Can you tell me about your work in relation to this subject or topic area?
4. In your experience, what helps / what hinders the family’s ability to provide emotional support for young people?
5. Factors internal to the family?
6. Factors external to the family?
7. How do these factors affect the family’s ability to provide emotional support for young people?
8. Which factors are most relevant in your work with young people and/or families? Why?
9. What do you think the impact of Covid-19 has been on the family as a source of emotional support for young people?
10. How do you use existing evidence in this area in your work? Existing evidence could include research or evaluation findings, for example, or quantitative or qualitative data etc.
11. What types of evidence in this area are most useful to you in your work? Why?
12. Are there particular theories, concepts, or models relating to the factors influencing the family as a source of emotional support for young people that you regularly use in your work? What / why?
13. What is your view on the quality of the current evidence in this area?
14. What recommendations would you make to improve the quality of evidence?
15. Where do you think there are gaps in the existing evidence on the factors that enable families to provide emotional support for young people to equip them during the transition to adulthood?
16. Do you think there are voices of any particular populations, family types, or family structures missing from the current evidence base? What / why?
17. Do you think there are any important topics or factors that haven’t been researched sufficiently yet? What / why?
18. Is there a need for more of a particular type of research study to be conducted or more of a particular type of data to be collected? What / why?
19. Why do you think there are gaps in the current evidence base?
20. Finally, what do you think should be funders’ and researchers’ next steps to expand the existing evidence base on the factors influencing the family as a source of emotional support for young people?
21. Are there any gaps in the existing evidence that it would be helpful, from your perspective, for researchers to address? What / why?
22. Are there any gaps in the existing evidence that it would be helpful, from your perspective, for funders to address? What / why?
23. How would this be useful for your work with young people or families?
